# Supplementary material for: Cargo receptor-assisted endoplasmic reticulum export of pathogenic α1-antitrypsin polymers
Source: Cell Rep. 2021 May 18;35(7):109144. doi: 10.1016/j.celrep.2021.109144 (PMC8149808; doi:10.1016/j.celrep.2021.109144)
Supplement: Document S1. Figures S1–S7 and Tables S2–S4 [file mmc1.pdf]

Cell Reports, Volume *35*

Supplemental information

Cargo receptor-assisted endoplasmic reticulum export of pathogenic α1-antitrypsin polymers

Adriana Ordóñez, Heather P. Harding, Stefan J. Marciniak, and David Ron

Supplementary Figure 1 (Fig. S1)

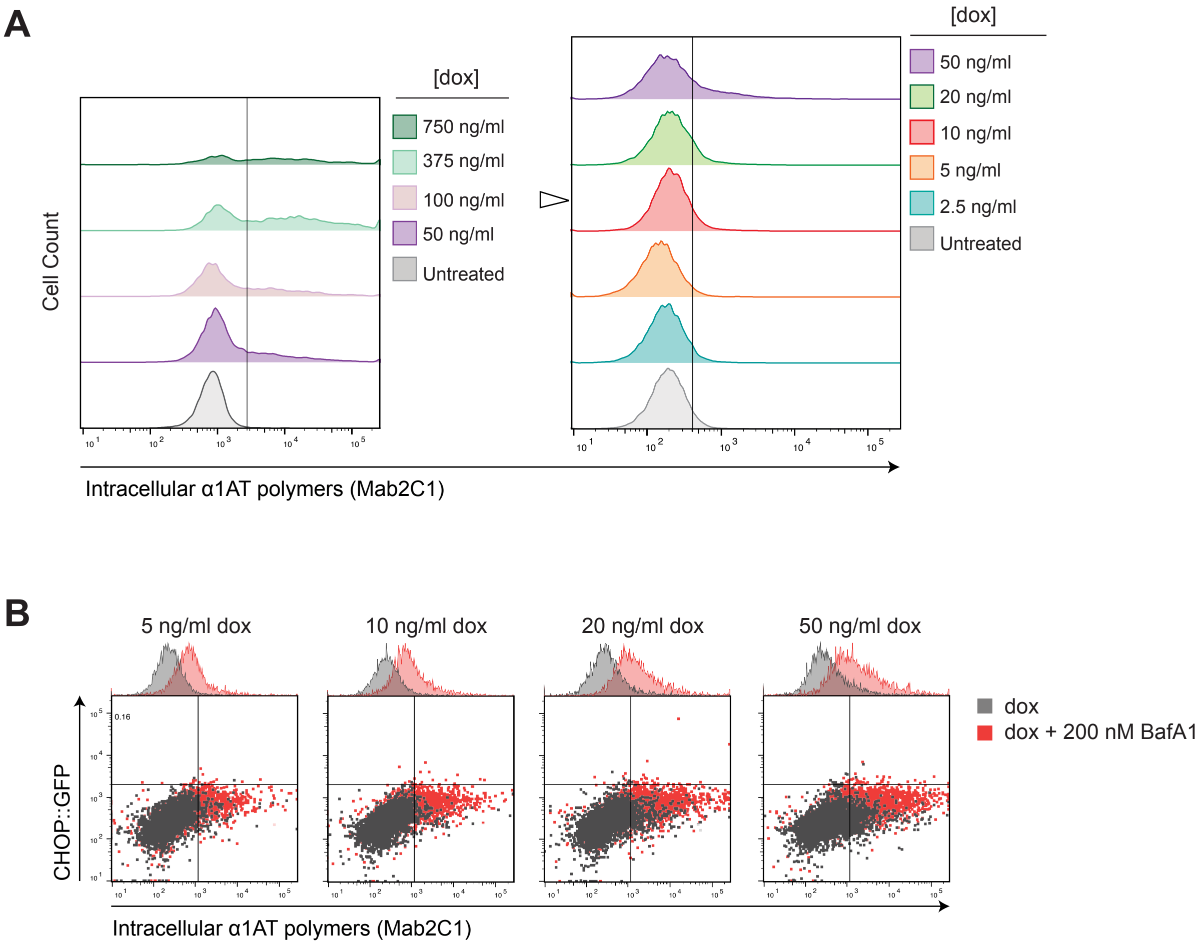

Fig. S1. Concentration-dependence of the response of CHO-K1 Tet-on cells to doxycycline and bafilomycinA1. Related to Figure 1.

(A) Flow cytometry analysis of the fluorescence intensity as a measure of intracellular α 1AT polymer levels (stained with Mab2C1) in CHO-K1 Tet-on_α1AT^{H334D}_Cas9 cells treated for 24 hrs with the indicated concentrations of doxycycline (dox). The left and right panels represent two independent experiments. The white arrowhead indicates the dox concentration used in the screen.

(B) Dual-channel flow cytometry of the UPR marker, *CHOP::GFP*, and intracellular levels of α1AT polymers in CHO-K1 Tet-on_α1AT^{H334D}_Cas9 cells treated for 24 hrs with the indicated concentration of dox in presence or absence of bafilomycinA1 (BafA1; 200 nM, added during the last 16 hrs). 5,000 cells were analyzed.

Supplementary Figure 2 (Fig. S2)

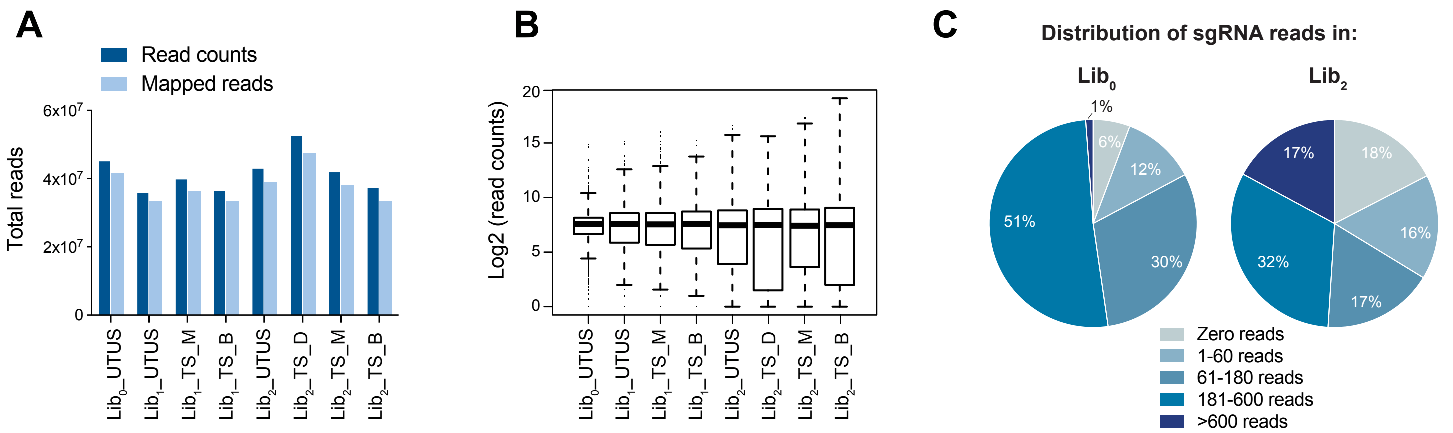

Fig. S2. Quality control data analysis of the CRISPR/Cas9 screen performed by MAGeCK. Related to Figure 2.

(A) Total read counts and reads mapped to the CHO library analysed by MAGeCK [UTUS: untreated (no doxycycline) and unsorted; TS: treated (plus doxycycline) and sorted; Lib_0: unenriched library) Lib_1: derivative enriched library 1; Lib_2: derivative enriched library 2; B: brightest; M: medium-bright; D: dull].

(B) Frequency distribution of sgRNA in each sample, showing the median-normalized read counts.

(C) Representation of sgRNAs in unsorted cells after infection with the unenriched genome-wide library (Lib_0) and enriched library (Lib_2) according to their read counts.

Supplementary Figure 3 (Fig. S3)

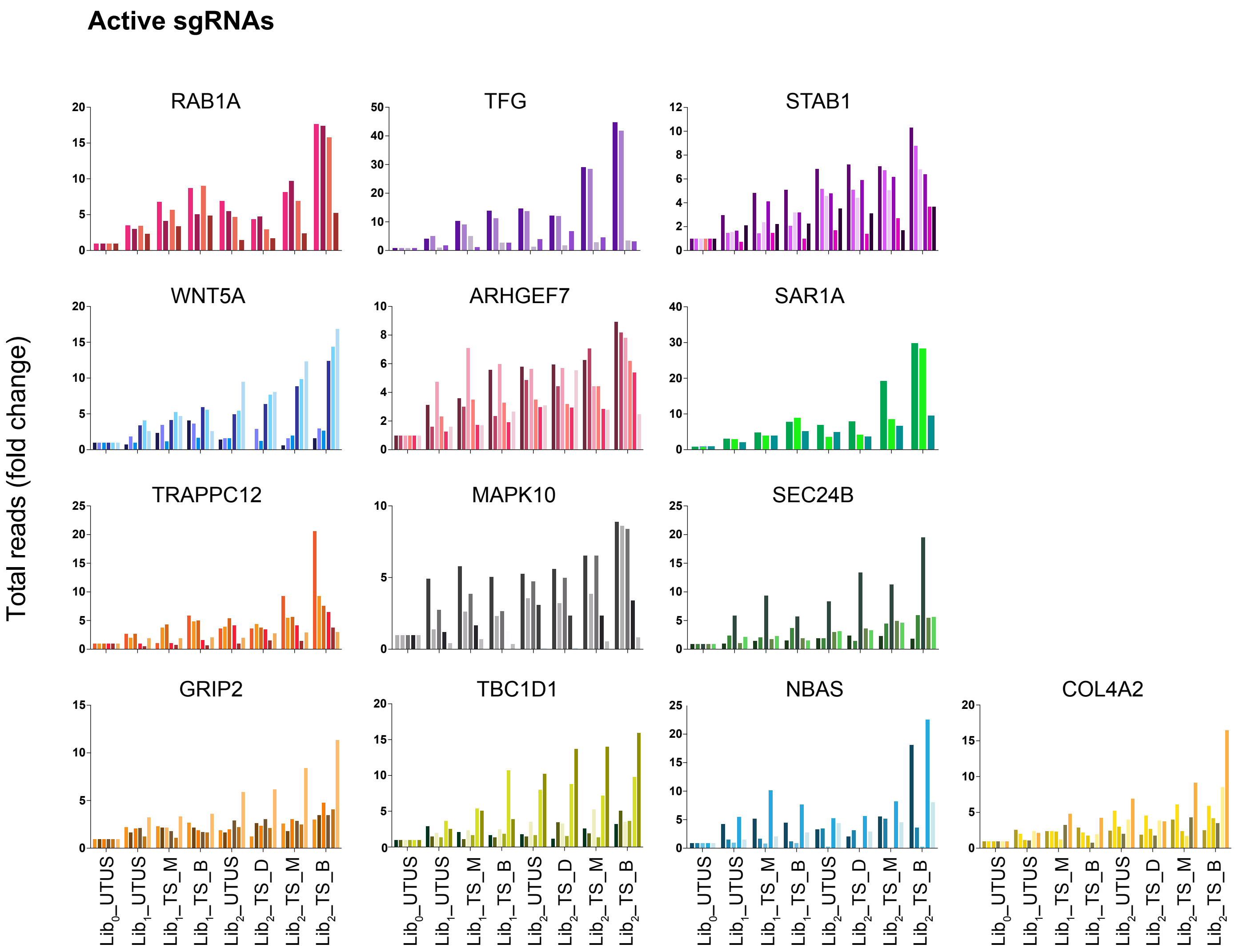

Fig. S3. The total reads counts for each active sgRNA targeting the remaining top 13 enriched genes included in the 'cargo loading into COPII-coated vesicle' cluster show an enrichment through the selection process. Related to Figure 2E.

Supplementary Figure 4 (Fig. S4)

A

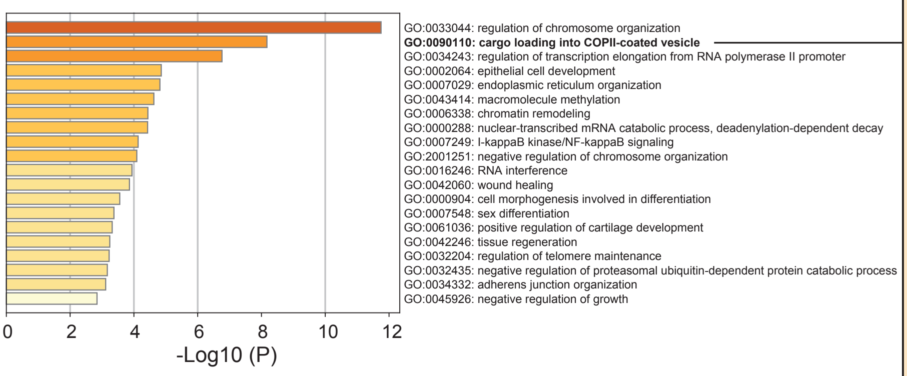

Gene	Description	Log2 (FC)
LMAN1	Lectin, mannose binding 1	*2.9204*
SEC23B	SEC23 homolog B, coat complex II component	*2.7843*
RAB1A	RAB1A, member RAS oncogene family	*2.3817*
TFG	Ttrafficking from ER to golgi regulator	*2.1409*
SAR1A	Secretion associated Ras related GTPase 1A	*2.032*
SURF4	Surfeit locus protein 4	*1.9554*
SEC24A	SEC24 homolog A, coat complex II component	*1.63*
WNT5A	Wnt family member 5A	*1.5715*
USE1	Unconventional SNARE in the ER 1	*1.5052*
BNIP1	BCL2 interacting protein 1	*1.429*
ALG12	ALG12 alpha-1,6-mannosyltransferase	*1.3852*
TRAPPC12	Trafficking protein particle complex 12	*1.3194*
PLEK	Pleckstrin	*1.1244*
IER3IP1	Immediate early response 3 interacting protein	*1.1125*
CASP3	Caspase 3	*1.0971*
STAB1	Stabilin 1	*1.0645*
SCFD2	Sec1 family domain containing 2	*0.97922*
COL4A2	Collagen type IV alpha 2 chain	*0.91986*
SEC24B	SEC24 homolog B, coat complex II component	*0.59458*
MON2	MON2 homolog, regulator of endosome-to-Golgi trafficking	*0.28108*

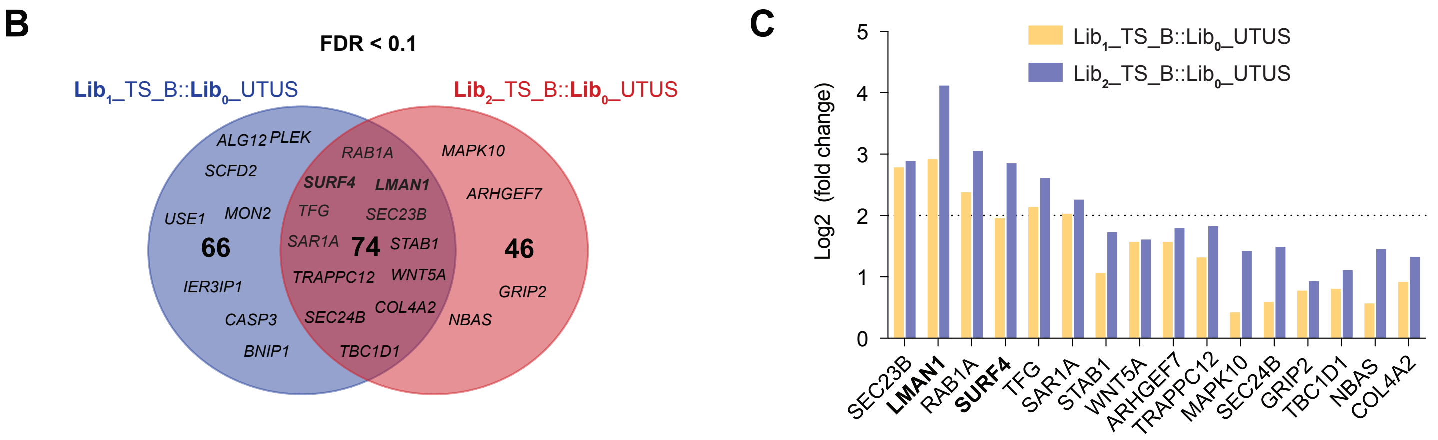

Fig. S4. Overlapping enrichment of specific sgRNAs targeting genes encoding components of the early secretory pathway in the first or second round of the CRISPR screen. Related to Figure 2.

(A) Gene ontology (GO) enrichment analysis of the top 140 hits identified in the CRISPR/Cas9 screen after the first round of enrichment (infection with Lib_1) and annotation of the 20 genes included in the GO term ‘cargo loading into COPII-coated vesicle’ indicating the corresponding Log2 (fold change) value for each gene.

(B) Venn diagram depicting unique and common upregulated top genes included in the ‘cargo loading into COPII-coated vesicle’ GO term, between the first (Lib_1) and second (Lib_2) round of enrichment after sorting.

(C) Histogram graph comparing the Log2 (fold change) values of the 16 genes included in the ‘cargo loading into COPII-coated vesicle’ cluster that were significantly enriched during the selection process after the first (Lib_1_TS_B::Lib_0_UTUS) and second round (Lib_2_TS_B::Lib_0_UTUS) of enrichment. Genes above the horizontal dashed line were enriched by a folded change of 4. LMAN1 and SURF4, the two cargo receptors selected for further investigation in our study, are in bold.

Supplementary Figure 5 (Fig. S5)

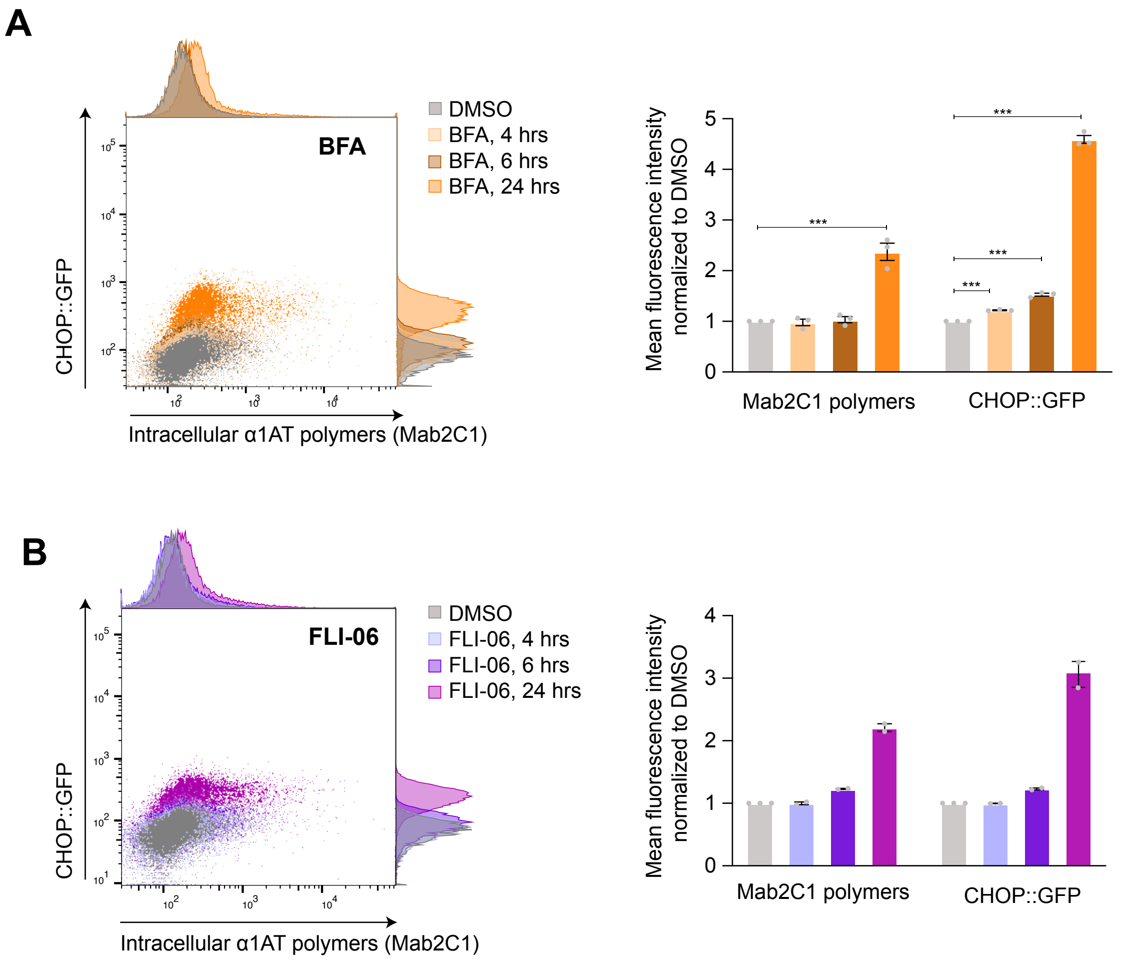

Fig. S5. Disruption of global endoplasmic reticulum-Golgi protein transport by brefeldin A (BFA) and FLI-06 increases the intracellular levels of α1-antitrypsin polymers and induces ER stress. Related to Figure 2.

(A) Dot plots of a representative dual-channel flow cytometry analysis of intracellular levels of α1AT polymers (Mab2C1) and *CHOP::GFP* reporter signal in CHO-K1 Tet-on_α1AT^{H334D} cells after treatment with brefeldin A (BFA). Cells were simultaneously induced with doxycycline (10ng/ml) and BFA (10 ug/ml) for 24 hrs, or induced with doxycycline for 24 hrs and BFA-treated for 4 and 6 hrs previous harvesting the cells. The bar graph shows the mean ± SEM of the Mab2C1-polymer and *CHOP::GFP* signal normalized to vehicle treated control cells (DMSO) from two independent experiments, one of them performed in duplicate (Unpaired t-test).

(B) As in "A" but plotting the intracellular levels of α1AT polymers and *CHOP::GFP* reporter signal after FLI-06 treatment (10 uM). The bar graph shows the mean ± SEM of one single experiment performed in duplicate.

Supplementary Figure 6 (Fig. S6)

A **Related to Fig. 5C**

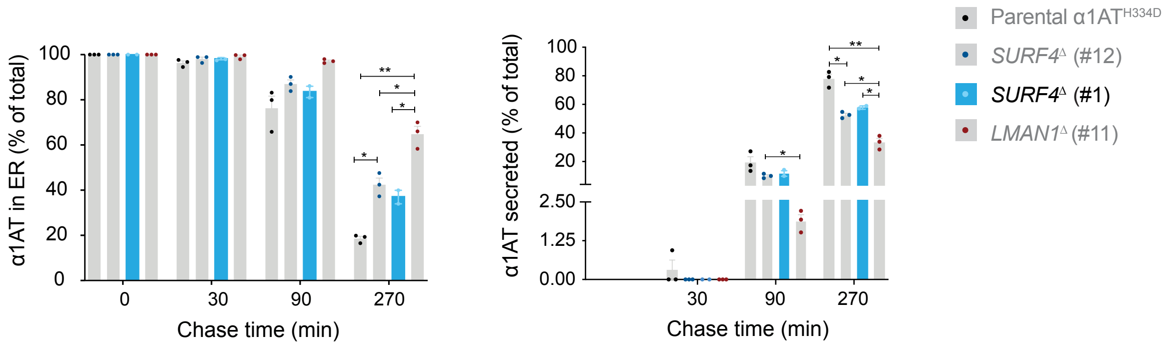

B **Related to Fig. 5D**

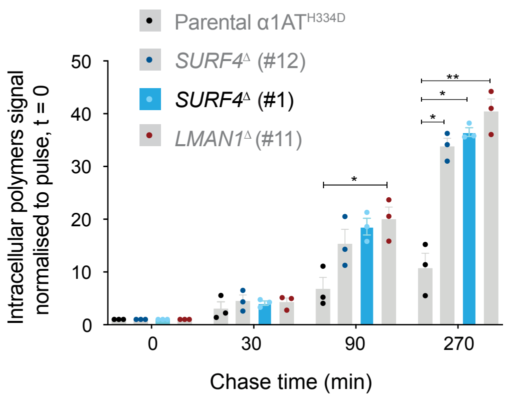

C **Related to Fig. 5E**

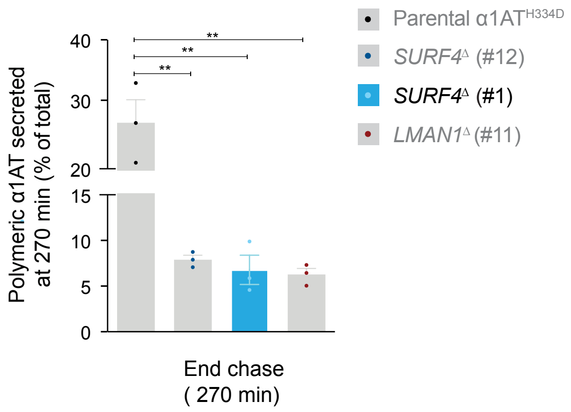

Fig. S6. Altered intracellular trafficking of α1-antitrypsin in an additional *SURF4*$^{\Delta}$ clone. Related to Figure 5.
Labelled α1AT was immunoprecipitated with a polyclonal antibody reactive with all α1AT forms or a monoclonal antibody selective for α1AT polymers from lysates of parental CHO-K1 Tet-on_α1AT^{H334D} cells and their *SURF4*$^{\Delta}$ and *LMAN1*$^{\Delta}$ derivatives or from the culture media supernatant.
(A) Related to Fig. 5C. Plots of the percentage of α1AT retained in the ER (left panel) or secreted into the media (right panel) at the indicated times. The additional *SURF4* disrupted clone [*SURF4*$^{\Delta}$ (#1)] is highlighted in blue and the other three genotypes (previously shown in Fig. 5C) are coloured in grey.
(B) Related to Fig. 5D. Plot of the intracellular polymer signal normalized to polymer α1AT signal at pulse end (t = 0) at the indicated times. The additional *SURF4*$^{\Delta}$ (#1) clone is highlighted in blue.
(C) Related to Fig. 5E. Plot of the percentage of α1AT polymers present in the media at 270 min. The additional *SURF4*$^{\Delta}$ (#1) clone is highlighted in blue.
All quantitative plots show the mean ± SEM of two or three independent experiments; $^{*}p<0.05$, $^{**}p<0.01$. Two-way (in 'A' and 'B') or one-way ANOVA (in 'C') followed by Tukey's post-hoc multiple comparison test.

Supplementary Figure 7 (Fig. S7)

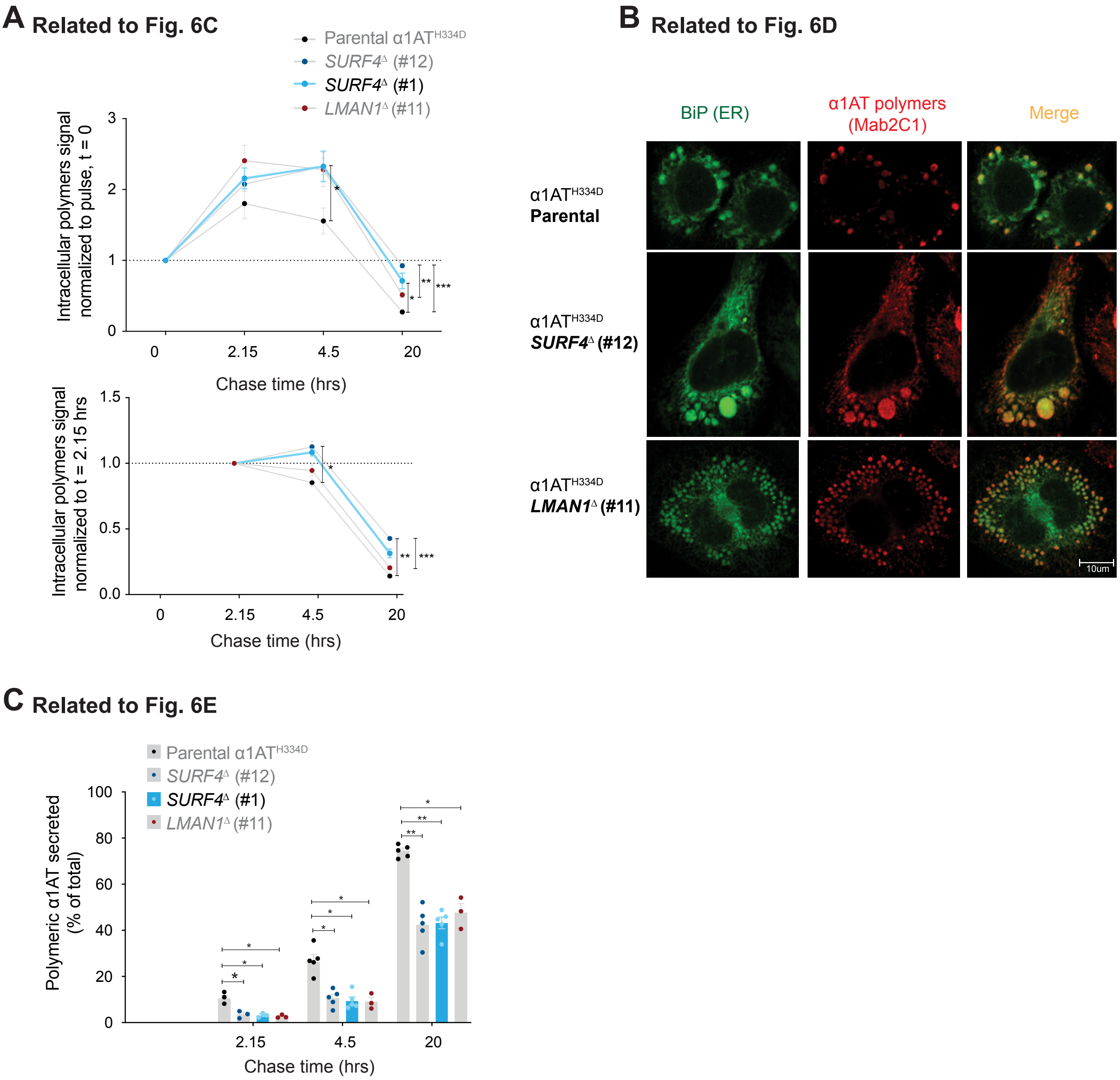

Fig. S7. SURF4 favours ER exit of α1-antitrypsin polymers in an additional $SURF4^{\Delta}$ clone. Related to Figure 6.

Labelled α1AT was immunoprecipitated with a monoclonal antibody selective for α1AT polymers from lysates of parental CHO-K1 Tet-on_α1AT^{H334D} cells and their *SURF4*$^{\Delta}$ and *LMAN1*$^{\Delta}$ derivatives or from the culture media supernatant.

(A) Related to Fig. 6C. Plots of the cell-associated α1AT polymer signal at the indicated times, normalized to the signal at pulse end [t = 0, (upper panel)] or at 2.15 hrs (bottom panel). The additional *SURF4* disrupted clone [*SURF4*$^{\Delta}$ (#1)] is highlighted in blue and the other three genotypes (previously showed in Fig. 6C) are coloured in grey.

(B) Related to Fig. 6D. Representative confocal immunofluorescence microscopy images of α1AT polymers (Mab2C1, red) together with an ER marker (BiP, green) in fixed parental CHO-K1 Tet-on_α1AT^{H334D} cells and their *SURF4*$^{\Delta}$ (#12) and *LMAN1*$^{\Delta}$ (#11) derivatives clones. α1AT expression was induced with 500 ng/ml doxycycline for 24 hrs.

(C) Related to Fig. 6E. Percentage of α1AT polymers present in the media at the indicated times. The additional *SURF4*$^{\Delta}$ (#1) clone is highlighted in blue.

All quantitative plots show the mean ± SEM of three to five independent experiments; *p<0.05, **p<0.01, ***p<0.001, ****p<0.0001. Two-way ANOVA test followed by Tukey's post-hoc multiple comparison test.

Supplemental Tables

Table S2: List of clones generated in this study. Related to STAR Methods.

Gene targeting	Cell line	Clone	Exon	Allele	Amino acid sequence (number shows amino acid position at which insert/deletion occurred)
SURF4	CHO-K1 Tet-on α1AT^{H334D}_CHOP::GFP	#12	5	1	….VTMR149in*
				2	….VTMR149in*
		#1	2	1	….DGIR44delSGVSNVTILTLPGAVATCWP HPLCSSTSWDS*
				2	ΔW45-Q47
	CHO-K1 Tet-on α1AT^{WT}_CHOP::GFP	#21	5	1	….VTMR149in*
				2	….VTMR149inLRGHPDRQAEGDQGWPPA LRLVRAPLSSTCNLEEGSCWS*
LMAN1	CHO-K1 Tet-on α1AT^{H334D}_CHOP::GFP	#11	9	1	….VSSL375delRRDLQERSGDPRAAWAGL STGTRYSCENPA*
				2	….VSSL375delKKRSPGEERGPQGSLGRS LNRN*
		#14	11	1	….QHPG433indelVYETTSALHGHQRAPAC REERY*
				2	….QHPG433inWHVVALAPVLVASGQHLVV DGVVLQKALRHDMVHIVVGQPVDVQFLVH VHIPPILQVVQVQLLVLQLGVFHGVFFQDL AAQLFDAXXDPXXSLAAVLLSRRRL*
	CHO-K1 Tet-on α1AT^{WT}_CHOP::GFP	#8	9	1	….VSSL375delRSPGEERGPQGSLGRSLN RN*
				2	….VSSL375delKKRSPGEERGPQGSLGRS LNRN*
SEC23B	CHO-K1 Tet-on α1AT^{H334D}_CHOP::GFP	#1	7	1	….KTP313delIPGTILKKIMHGS*
				2	….KTP313delIPGTILKKIMHGS*

Table S3: List of sgRNAs and oligonucleotides used in this study. Related to STAR Methods.

Lab ID	Name	Sequence 5' – 3'	Comment	Reference
2486	cgLman1_g1_e11_1S	CACCGCTCATAGACGCCTG CAGAGC	CRISPR-Cas9 guide targeting chinese hamster Lman1 exon 11	This study
2487	cgLman1_g1_e11_2A S	AAACGCTCTGCAGGCGTCT ATGAGC	CRISPR-Cas9 guide targeting chinese hamster Lman1 exon 11	This study
2488	cgLman1_g2_e9_1S	CACCGCCTGGAGATCTCTT CTGTCA	CRISPR-Cas9 guide targeting chinese hamster Lman1 exon 9	This study
2489	cgLman1_g2_e9_2A S	AAACTGACAGAAGAGATCT CCAGGC	CRISPR-Cas9 guide targeting chinese hamster Lman1 exon 9	This study
2490	cgSurf4_g1_e5_1S	CACCGCTTAGGGGAGCTCT CACGCA	CRISPR-Cas9 guide targeting chinese hamster Surf4 exon 5	This study
2491	cgSurf4_g1_e5_2AS	AAACTGCGTGAGAGCTCCC CTAAGC	CRISPR-Cas9 guide targeting chinese hamster Surf4 exon 5	This study
2492	cgSurf4_g2_e2_1S	CACCGCATCCGCATGTGGT TTCAG	CRISPR-Cas9 guide targeting chinese hamster Surf4 exon 2	This study
2493	cgSurf4_g2_e2_2AS	AAACCTGAAACCACATGCG GATGC	CRISPR-Cas9 guide targeting chinese hamster Surf4 exon 2	This study
2494	cgSec23b_g1_e7_1S	CACCGATATCGTGCCAGGA ACGAAT	CRISPR-Cas9 guide targeting chinese hamster Sec23b exon 7	This study
2495	cgSec23b_g1_e7_2A S	AAACATTCGTTCCTGGCAC GATATC	CRISPR-Cas9 guide targeting chinese hamster Sec23b exon 7	This study
2496	cgSec23b_g2_e13_1 S	CACCGCAGTCTTGATGGCA CGGCT	CRISPR-Cas9 guide targeting chinese hamster Sec23b exon 13	This study
2497	cgSec23b_g2_e13_2 AS	AAACAGCCGTGCCATCAAG ACTGC	CRISPR-Cas9 guide targeting chinese hamster Sec23b exon 13	This study
2547	cgSurf4_e2_1S	ACCAAGCAGTACCTGCCTC A	for sequencing CRISPR mutants made in the cgSurf4 locus	This study
2548	cgSurf4_e2_2AS	ACACAAAGGATGAGGCCAA C	for sequencing CRISPR mutants made in the cgSurf4 locus	This study
2549	cgSurf4_e5_1S	GAGGTTTGCTGCTGCTCTT G	for sequencing CRISPR mutants made in the cgSurf4 locus	This study
2550	cgSurf4_e5_2AS	AGCTGGCATCAAAGTGAAG G	for sequencing CRISPR mutants made in the cgSurf4 locus	This study
2516	cgLman1_e11_1S	GAACTCCATGAGTGAAACA GTCC	for sequencing CRISPR mutants made in the cgLman1 locus	This study
2517	cgLman1_e11_2AS	ATGTTGCGCTGAGCAAGG	for sequencing CRISPR mutants made in the cgLman1 locus	This study
2518	cgLman1_e9_1S	CGATCGCGAGCTAAGACAA G	for sequencing CRISPR mutants made in the cgLman1 locus	This study
2519	cgLman1_e9_2AS	CTGGAGCATTTTGAGGGAA C	for sequencing CRISPR mutants made in the cgLman1 locus	This study
2528	cgSec23b_e7_1S	GGATCATGCTGTTCACTGG A	for sequencing CRISPR mutants made in the cgSec23b locus	This study
2529	cgSec23b_e7_2AS	AGTGACAGCTGGAATCCAC A	for sequencing CRISPR mutants made in the cgSec23b locus	This study
2182	sgRNA_outer_MluI_s hort_F	CAGCAGAGATCCAGTTTGG TTAGTACC	primer for PCR of pKLV CHO_CRISPR library for recloning in UK1789	This study
1432	P5-sgRNA_inner_F	AATGATACGGCGACCACCG AGATCTACACTCTCTTGTGG AAAGGACGAAACACCG	primer for barcoding and adapting lentiGuide PCR products from CRISPR library screening for NGS	Harding et al., 2019
1434	sgRNA_outer_short_ F	GCTTACCGTAACTTGAAAGT ATTTCG	primer for barcoding and adapting lentiGuide PCR products from CRISPR library screening for NGS	Harding et al., 2019
1435	Illumina-sgRNA_seq	ACACTCTCTTGTGGAAAGG ACGAAACACCG	PAGE purified primer for NGS of PCR products from CRISPR library screening	Harding et al., 2019
1758	sgRNA_outer_short_ R2	GAATGTGTGCGAGGCCAGA G	primer for 1st round PCR of pKLV CHO_CRISPR library for NGS sequencing	Harding et al., 2019

1759	pKLV_NEBNXT01	CAAGCAGAAGACGGCATACGAGATCGTGATGTGACTGGAGTTCAGACGTGTGCTCTTCCGATCTGAGGCCACTTGTGTAGCGCCAAG	primer for barcoding and adapting pKLV CHO_CRISPR PCR products for NGS	Harding et al., 2019
1760	pKLV_NEBNXT02	CAAGCAGAAGACGGCATACGAGATACATCGGTGACTGGAGTTCAGACGTGTGCTCTTCCGATCTGAGGCCACTTGTGTAGCGCCAAG	primer for barcoding and adapting pKLV CHO_CRISPR PCR products for NGS	Harding et al., 2019
1761	pKLV_NEBNXT03	CAAGCAGAAGACGGCATACGAGATTGCCTAAGTGACTGGAGTTCAGACGTGTGCTCTTCCGATCTGAGGCCACTTGTGTAGCGCCAAG	primer for barcoding and adapting pKLV CHO_CRISPR PCR products for NGS	Harding et al., 2019
1762	pKLV_NEBNXT04	CAAGCAGAAGACGGCATACGAGATTGGTCAGTGACTGGAGTTCAGACGTGTGCTCTTCCGATCTGAGGCCACTTGTGTAGCGCCAAG	primer for barcoding and adapting pKLV CHO_CRISPR PCR products for NGS	Harding et al., 2019
1763	pKLV_NEBNXT05	CAAGCAGAAGACGGCATACGAGATCACTGTGTGACTGGAGTTCAGACGTGTGCTCTTCCGATCTGAGGCCACTTGTGTAGCGCCAAG	primer for barcoding and adapting pKLV CHO_CRISPR PCR products for NGS	Harding et al., 2019
1764	pKLV_NEBNXT06	CAAGCAGAAGACGGCATACGAGATTATTGGCGTGACTGGAGTTCAGACGTGTGCTCTTCCGATCTGAGGCCACTTGTGTAGCGCCAAG	primer for barcoding and adapting pKLV CHO_CRISPR PCR products for NGS	Harding et al., 2019
1765	pKLV_NEBNXT07	CAAGCAGAAGACGGCATACGAGATTGATCTGGTGACTGGAGTTCAGACGTGTGCTCTTCCGATCTGAGGCCACTTGTGTAGCGCCAAG	primer for barcoding and adapting pKLV CHO_CRISPR PCR products for NGS	Harding et al., 2019
1766	pKLV_NEBNXT08	CAAGCAGAAGACGGCATACGAGATTTCAAGTGTGACTGGAGTTCAGACGTGTGCTCTTCCGATCTGAGGCCACTTGTGTAGCGCCAAG	primer for barcoding and adapting pKLV CHO_CRISPR PCR products for NGS	Harding et al., 2019
1767	pKLV_NEBNXT09	CAAGCAGAAGACGGCATACGAGATTCTGATCGTGACTGGAGTTCAGACGTGTGCTCTTCCGATCTGAGGCCACTTGTGTAGCGCCAAG	primer for barcoding and adapting pKLV CHO_CRISPR PCR products for NGS	Harding et al., 2019
1768	pKLV_NEBNXT10	CAAGCAGAAGACGGCATACGAGATAAGCTAGTGACTGGAGTTCAGACGTGTGCTCTTCCGATCTGAGGCCACTTGTGTAGCGCCAAG	primer for barcoding and adapting pKLV CHO_CRISPR PCR products for NGS	Harding et al., 2019
1769	pKLV_NEBNXT11	CAAGCAGAAGACGGCATACGAGATTGTAGCCGTGACTGGAGTTCAGACGTGTGCTCTTCCGATCTGAGGCCACTTGTGTAGCGCCAAG	primer for barcoding and adapting pKLV CHO_CRISPR PCR products for NGS	Harding et al., 2019
2606	cgSurf4_exon4_6FAM_2AS	[6FAM]AGCTGGCATCAAAGTGAAGG	oligo 2550 with 5'-[6FAM] for screening for efficient CRISPRs	This study
2607	cgSurf4_exon1_6FAM_2AS	[6FAM]ACACAAAGGATGAGGCCAAC	oligo 2548 with 5'-[6FAM] for screening for efficient CRISPRs	This study
2665	cgLman1_exon10_6FAM_2AS	[6FAM]ATGTTGCGCTGAGCAAGG	oligo 2517 with 5'-[6FAM] for screening for efficient CRISPRs	This study
2666	cgLman1_exon8_6FAM_2AS	[6FAM]CTGGAGCATTTTGAGGGAAC	oligo 2519 with 5'-[6FAM] for screening for efficient CRISPRs	This study
1402	EGFP_guide1_1S	CACCGGGCGAGGAGCTGTTCACCG	CRISPR-Cas9 guide targeting EGFP	This study
1403	EGFP_guide1_2AS	AAACCGGTGAACAGCTCCTCGCCC	CRISPR-Cas9 guide targeting EGFP	This study

Table S4: List of Recombinant DNA used in this study. Related to STAR Methods.

Lab ID	Plasmid name	Description	Reference
UK1610	pSpCas9(BB)-2A-mCherry	Modified pSpCas9(BB)-2A vector to express mCherry together with guide RNA & Cas9	Amin-Wetzel N et al., 2017
UK1700	pMD2.G	Addgene plasmid 12259, lentiviral packaging helper, (VSVG)	Unpublished, gift from Didier Trono
UK1701	psPAX2	Addgene plasmid 12260, next gen lentiviral packaging helper	Unpublished, gift from Didier Trono
UK1702	LentiGuide-puro	Addgene plasmid 52963	Sanjana et al., 2014, gift from Feng Zhang
UK1714	Lenti-Cas9	Lenti-Cas9 in which 2TA-blast sequence is removed to make a lenti-Cas9 without resistance selection marker	This study
UK1717	EGFPsgRNA_lentiGuide-Puro	Lentiviral vector expressing EGFP CRISPR guides without expression of Cas9	This study
UK1789	pKLV-U6gRNA(BbsI)-PGKpuro2ABFP	Addgene 50946, BFP-2A-Puro tagged gRNAvector	Koike-Yusa et al., 2014, gift from Kosuke Yusa
UK1857	cgHSPA5_g1_pSpCas(BB)-2A-mCherry	mCherry-tagged CRISPR plasmid (UK1610) for targeting hamster HSPA5 (BiP)	Preissler et al., 2017
UK1858	cgHSPA5_g2_pSpCas(BB)-2A-mCherry	mCherry-tagged CRISPR plasmid (UK1610) for targeting hamster HSPA5 (BiP)	Preissler et al., 2017
UK2561	pKLV-CHO_libA-PGKpuro2ABFP (Library0)	CHO CRISPR KO library of 125030 selected guides for whole genome CRISPR screening	Unpublished
UK2321	pKLV-α1AT derivative enriched CHO library1 (MluI_BamHI)-PGKpuro2ABFP	CHO CRISPR KO derivative library 1 (Lib1) for α1AT polymer enrichment_Brightest population-After first sorting	This study
UK2378	pKLV-α1AT derivative enriched CHO library2 (MluI_BamHI)-PGKpuro2ABFP	CHO CRISPR KO derivative library 2 (Lib2) for α1AT polymer enrichment_Brightest population-After second sorting	This study
UK2501	cgLman1_g1_exon 11_pSpCas9(BB)-2A-mCherry	mCherry-tagged CRISPR plasmid (UK1610) targeting cgLMAN1_guide 1	This study
UK2502	cgLman1_g2_exon 9_pSpCas9(BB)-2A-mCherry	mCherry-tagged CRISPR plasmid (UK1610) targeting cgLMAN1_guide 2	This study
UK2503	cgSurf4_g1_exon 5_pSpCas9(BB)-2A-mCherry	mCherry-tagged CRISPR plasmid (UK1610) targeting cgSURF4_guide 1	This study
UK2504	cgSurf4_g2_exon 2_pSpCas9(BB)-2A-mCherry	mCherry-tagged CRISPR plasmid (UK1610) targeting cgSURF4_guide 2	This study
UK2505	cgSec23b_g1_exon 7_pSpCas9(BB)-2A-mCherry	mCherry-tagged CRISPR plasmid (UK1610) targeting cgSEC23b_guide 1	This study
UK2506	cgSec23b_g2_exon 13_pSpCas9(BB)-2A-mCherry	mCherry-tagged CRISPR plasmid (UK1610) targeting cgSEC23b_guide 2	This study
UK2549	FLAG-tagged SURF4 [pNLF-FLAG-SURF4-puro)	FLAG-tagged SURF4 [pNLF-FLAG-SURF4-puro)	Emmer et al., 2018, gift from David Ginsburg
UK2622	pNLF-H7-SURF4-puro	Mammalian expression plasmid 7xHis N-term tagged SURF4	This study
