# Supplementary material for: Cargo receptor-assisted endoplasmic reticulum export of pathogenic α1-antitrypsin polymers
Source: Cell Rep. 2021 May 18;35(7):109144. doi: 10.1016/j.celrep.2021.109144 (PMC8149808; doi:10.1016/j.celrep.2021.109144)
Supplement: Document S2. Article plus supplemental information [file mmc3.pdf]

Cell Reports

Article

Cargo receptor-assisted endoplasmic reticulum export of pathogenic α1-antitrypsin polymers

Graphical abstract

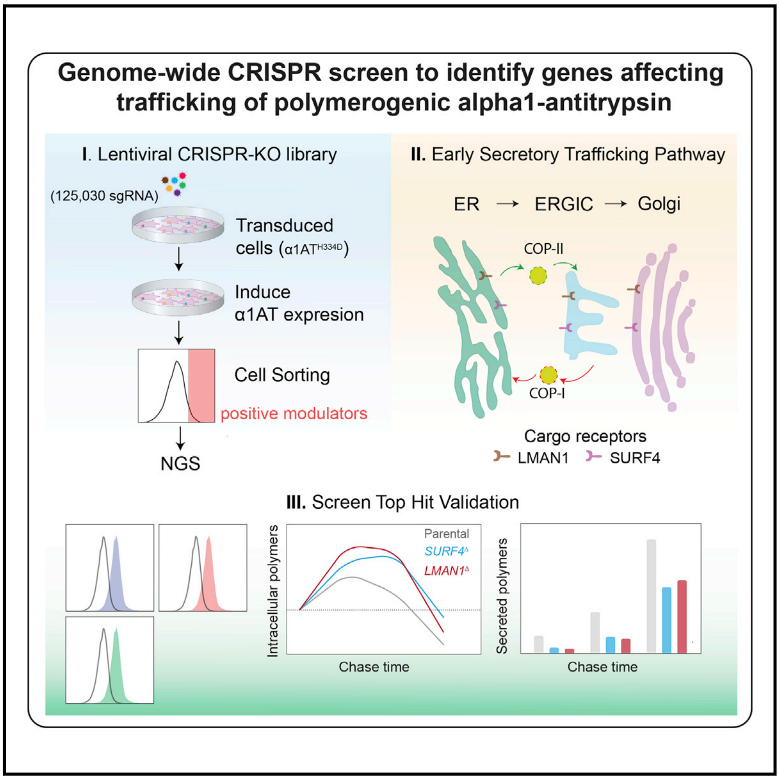

Highlights

- Genome-wide CRISPR screen for genes affecting trafficking of polymerogenic α1AT
- CRISPR enrichment based on recovery of DNA from phenotypic selected fixed cells
- Early secretory pathway as the strongest modifier of α1AT intracellular polymers
- LMAN1 and SURF4 regulate the export of monomeric and polymeric α1AT out of the ER

Authors

Adriana Ordóñez, Heather P. Harding, Stefan J. Marciniak, David Ron

Correspondence

aog23@cam.ac.uk

In brief

Ordóñez et al. report that ER cargo receptors regulate α1AT trafficking and modulate polymeric α1AT accumulation by controlling the concentration of monomers and promoting polymers ER exit. The latter implicates conventional intracellular trafficking in the early steps of polymer secretion and sheds light on the biogenesis of pro-inflammatory circulating polymers.

Ordóñez et al., 2021, Cell Reports *35*, 109144
May 18, 2021 © 2021 The Authors.
https://doi.org/10.1016/j.celrep.2021.109144

CellPress

Cell Reports

CellPress OPEN ACCESS

Article

Cargo receptor-assisted endoplasmic reticulum export of pathogenic α1-antitrypsin polymers

Adriana Ordóñez,[1,2,*] Heather P. Harding,[1] Stefan J. Marciniak,[1] and David Ron[1]
[1]Cambridge Institute for Medical Research (CIMR), University of Cambridge, Cambridge Biomedical Campus, The Keith Peters Building, Cambridge CB2 0XY, UK
[2]Lead contact
*Correspondence: aog23@cam.ac.uk
https://doi.org/10.1016/j.celrep.2021.109144

SUMMARY

Circulating polymers of α1-antitrypsin (α1AT) are neutrophil chemo-attractants and contribute to inflammation, yet cellular factors affecting their secretion remain obscure. We report on a genome-wide CRISPR-Cas9 screen for genes affecting trafficking of polymerogenic α1AT^{H334D}. A CRISPR enrichment approach based on recovery of single guide RNA (sgRNA) sequences from phenotypically selected fixed cells reveals that cells with high-polymer content are enriched in sgRNAs targeting genes involved in "cargo loading into COPII-coated vesicles," where "COPII" is coat protein II, including the cargo receptors lectin mannose binding1 (LMAN1) and surfeit protein locus 4 (SURF4). *LMAN1*- and *SURF4*-disrupted cells display a secretion defect extending beyond α1AT monomers to polymers. Polymer secretion is especially dependent on SURF4 and correlates with a SURF4-α1AT^{H334D} physical interaction and with their co-localization at the endoplasmic reticulum (ER). These findings indicate that ER cargo receptors co-ordinate progression of α1AT out of the ER and modulate the accumulation of polymeric α1AT not only by controlling the concentration of precursor monomers but also by promoting secretion of polymers.

INTRODUCTION

α1-antitrypsin (α1AT) (*SERPINA1*) is a glycoprotein synthesized primarily in hepatocytes and secreted as a monomer into blood to constitute the most abundant serine protease inhibitor (SERPIN) in circulation. Its main function is to inhibit neutrophil elastase in lungs defending against excessive tissue degradation by the endogenous protease-enzyme activity (Carrell and Lomas, 2002).

Missense variants in *SERPINA1*, including the most common Z variant (E342K), perturb the stability and conformation of α1AT monomers, resulting in their intracellular retention and formation of ordered and pathogenic polymers that accumulate within the lumen of the endoplasmic reticulum (ER) of hepatocytes. Intracellular retention is the basis of plasma α1AT deficiency underlying early-onset emphysema (Gooptu et al., 2014). Accumulation of polymers within liver cells is also associated with a toxic gain-of-function that predisposes to neonatal hepatitis and hepatocellular carcinoma (Eriksson et al., 1986). Interestingly, only 10%–15% of patients develop severe liver pathology, suggesting variation in the handling of intracellular polymers (Wu et al., 1994).

Although α1AT polymers are most abundant intracellularly, polymers have also been identified in circulation (Tan et al., 2014), in tissues, in the skin and kidney of α1AT-deficient patients with panniculitis (Gross et al., 2009) or vasculitis (Morris et al., 2011), and in bronchoalveolar lavage fluid of patients with lung disease (Morrison et al., 1987). *In vitro* (Mulgrew et al., 2004) and *in vivo* (Mahadeva et al., 2005) studies implicate extracellular polymers as chemo-attractants for human neutrophils that could contribute to inflammation and lung damage and less common extra-pulmonary manifestations of α1AT deficiency (Gooptu and Lomas, 2008).

Despite its importance to disease development, the processing and fate of intracellular polymers remain poorly understood. Both autophagy and ER-associated degradation (ERAD) have been implicated in their clearance (Kroeger et al., 2009). Less is known about how polymers reach the extracellular compartment. This has long been thought to be the result of either polymer release from dying cells or polymerization of mutant α1AT secreted as monomers. Recently, studies of plasma of α1AT-deficient patients before and after liver transplant (Tan et al., 2014) and cellular models suggest that circulating polymers are more likely to arise from secretion of pre-formed polymers rather than polymerization extracellularly (Fra et al., 2016). Notably, levels of polymers in plasma from α1AT-deficient patients do not increase after incubation at 37°C for 3 days (Fra et al., 2016). This observation suggests that plasma levels of mutant polymerogenic α1AT (which are typically 10%–15% the levels found in normal individuals) are below the threshold for aggregation. However, the processes underlying polymer secretion remain largely unknown.

© 2021 The Authors.
This is an open access article under the CC BY license (http://creativecommons.org/licenses/by/4.0/).

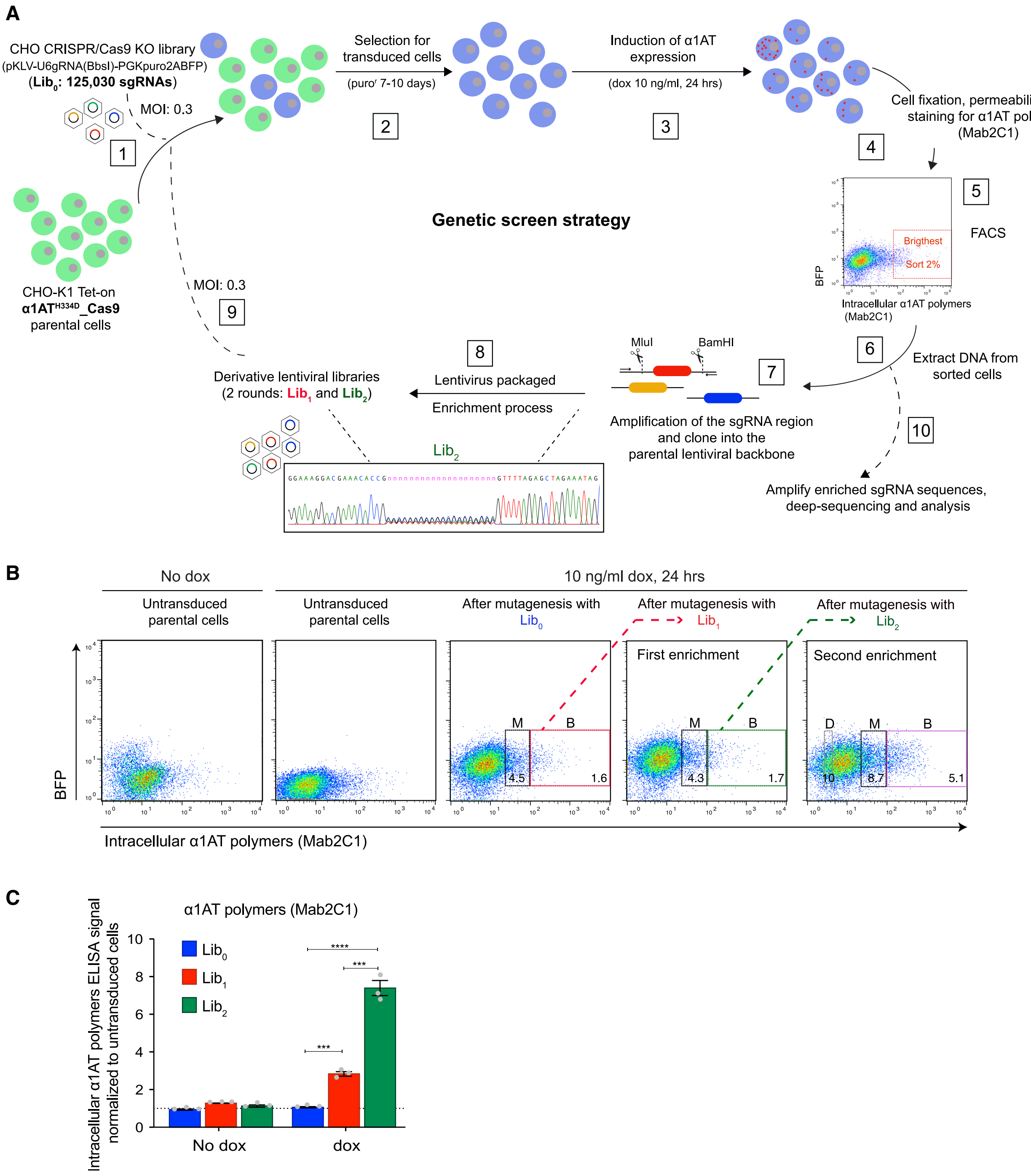

Figure 1. CRISPR-Cas9 screen to identify modifiers of intracellular levels of α1-antitrypsin (α1AT) polymers

(A) Workflow of a genome-wide CRISPR-Cas9 knockout (KO) screen. CHO-K1 cells expressing Cas9 and a Tet-inducible allele of α1AT^{H334D} were transduced at low multiplicity of infection (MOI: 0.3) with a lentiviral library of sgRNAs targeting the whole CHO genome (Lib_0) [1]. Transduced cells were selected for the presence of the puromycin resistance marker [2]. Expression of the α1AT^{H334D} transgene was induced with doxycycline (dox) [3]. Cells were fixed and stained for polymeric α1AT using the polymer-specific monoclonal antibody 2C1 (Mab2C1) [4] and FACS sorted based on signal intensity [5]. Genomic DNA was extracted from pools of cells with the highest level of polymer signal (“brightest”) [6] and used to amplify enriched sgRNA sequences to create new lentiviral libraries (Lib_1 and Lib_2). Sanger sequencing indicates the presence of sgRNA sequence diversity in the new lentiviral Lib_2 [7 and 8]. The selection cycle was repeated [9], and at its conclusion [10] genomic DNA from the selected cells was prepared for high-throughput sequencing and analysis of the successively enriched sgRNA sequences.

(legend continued on next page)

Here, we performed a forward genetic screen to identify components affecting the intracellular levels of a highly polymerogenic α1AT variant, the King's mutant (H334D) (Miranda et al., 2010). Our observations indicate that α1AT polymers can be secreted from the cells by the canonical secretory pathway and identify lectin mannose binding1 (LMAN1) and surfeit protein locus 4 (SURF4) as cargo receptors involved in the trafficking of monomeric and polymeric α1AT.

RESULTS

Flow cytometry-based assay to monitor intracellular α1AT polymers

To identify genes that modify intracellular levels of α1AT polymers, we developed a quantitative fluorescence-activated cell sorting (FACS)-compatible readout for the abundance of intracellular polymers using the well-described α1AT polymer-specific monoclonal antibody 2C1 (Mab2C1) (Miranda et al., 2010) in a previously characterized CHO-K1 cell line (Ordóñez et al., 2013). These cells express the polymerogenic variant (H334D) of α1AT, under control of a tetracycline-inducible (Tet-on) promoter that enables tight regulation of α1AT expression (Figure S1A). A derivative CHO-K1 Tet-on_α1AT^{H334D} clone that stably expresses Cas9 and maintained parental regulation of Tet-inducible α1AT^{H334D} expression was selected for screening.

To favor an experimental system that could respond to genetic perturbations with an increase in intracellular α1AT^{H334D} polymers, we treated cells with a range of concentrations of doxycycline in the absence or presence of BafilomycinA1, an inhibitor of lysosomal activity. BafilomycinA1 enhances accumulation of α1AT polymers (Kroeger et al., 2009) and proved useful in exploring the dynamic range of the assay. Doxycycline at 5–50 ng/mL was associated with low basal levels of Mab2C1 staining that increased conspicuously upon BafilomycinA1 treatment, suggesting a suitable assay window for the screen (Figure S1B).

A genome-wide screen identifies a set of genes affecting the intracellular itinerary of polymerogenic α1AT

CHO-K1 Tet-on_α1AT^{H334D}_Cas9 cells were initially transduced with a genome-wide CRISPR-Cas9 knockout library (Lib_0) comprising 125,030 single guide RNAs (sgRNAs) (Figure 1A) (~520× coverage). α1AT^{H334D} expression was then induced with doxycycline, followed 24 h later by fixation, permeabilization, and staining with the Mab2C1 primary antibody. Cells were FACS sorted into three bins based on Mab2C1-dependent fluorescence intensity: "brightest," "medium-bright," and "dull" (Figure 1B).

Cell fixation, required to detect intracellular polymers, precluded conventional enrichment schemes through successive rounds of phenotypic selection and expansion of the pooled cells. To circumvent this impasse, we implemented an approach based on recovery of sgRNA sequences from phenotypically selected cell populations (Figures 1A and 1B). Genomic DNA from the "brightest"-sorted cells was extracted, and fragments covering integrated sgRNA sequences were PCR amplified and used to generate a derivative CRISPR library (Figure 1A, lower segment). The derivative library (Lib_1), enriched in viral particles bearing phenotype-linked sgRNA sequences, was transduced into parental CHO-K1 Tet-on_α1AT^{H334D}_Cas9 cells followed by further phenotypic selection and generation of a second, enriched derivative library (Lib_2; Figure 1B). Transduction with Lib_0, Lib_1, and Lib_2 progressively increased intracellular α1AT polymers, as assessed by FACS (Figure 1B) and ELISA (Figure 1C).

Next, genomic DNA, pooled from sorted cells in the different bins at different stages of the phenotypic enrichment process and from unsorted control cells, was subjected to high-throughput sequencing (next-generation sequencing [NGS]) and MAGeCK bioinformatics analysis (Li et al., 2014) to determine sgRNA sequence enrichment and the corresponding gene ranking list (Table S1). Quality control based on sgRNA sequence read counts showed that over 90% of the reads mapped to the libraries (Figure S2A). Distribution of normalized read counts indicated that after successive rounds of positive phenotypic selection, the diversity of sgRNA species declined from libraries Lib_0 to Lib_2, with increasing percentage of sgRNAs with zero read counts and sgRNA with very high counts (Figures S2B and S2C).

Gene Ontology (GO) analysis of the most significantly enriched genes in the "brightest" Mab2C1-stained cells (with a false discovery rate [FDR] < 0.1) after infection with Lib_2 revealed that "regulation of chromosome organization" was the strongest selected GO term (Figure 2A). This cluster, thought to reflect the indirect effects of altered transcriptional regulation on polymer levels, was not further considered. The second highly represented cluster was "cargo loading into COPII-coated vesicle," where "COPII" is coat protein II, which included 16 genes that were significantly enriched during the selection process (Figures 2A and 2B; Figure S3). These encode components of the COPII complex that initiates vesicle budding at the ER (*SEC23B*, *SAR1A*, and *SEC24B*), non-COPII proteins important to vesicle formation (*RAB1A*, *TFG*, *TRAPPC12*, and *MAPK10*) (D'Arcangelo et al., 2013), and two cargo receptors with a known role in protein transport from the ER to Golgi apparatus (*LMAN1* and *SURF4*) (Gomez-Navarro and Miller, 2016). Albeit most of these genes were also significantly enriched in the "brightest" Mab2C1-stained cells after infection with Lib_1 (Figure S4), a second round of enrichment with Lib_2 showed a selection process (Figures S3 and S4C) and strongly reassured the important role of the "cargo loading into COPII-coated vesicle" cluster in the intracellular levels of α1AT polymers. In addition, the protein-

(B) Dual-channel flow cytometry of intracellular levels of α1AT polymers (stained with Mab2C1) and blue fluorescent protein (BFP; transduction marker) in α1AT^{H334D}-expressing cells before and after transduction with Lib_0 (unenriched library) and successively enriched Lib_1 and Lib_2. The boxed areas include the cells sorted for genomic analysis: "brightest" (B), "medium-bright" (M), and "dull" (D).

(C) Intracellular α1AT polymer signals quantified by sandwich ELISA of unsorted cells, transduced with Lib_0, Lib_1, and Lib_2, respectively, in the presence or absence of dox (10 ng/mL, 24 h). Shown is the mean ± SEM normalized to untransduced cells of three independent experiments. ***$p < 0.001$, ****$p < 0.0001$, unpaired t test.

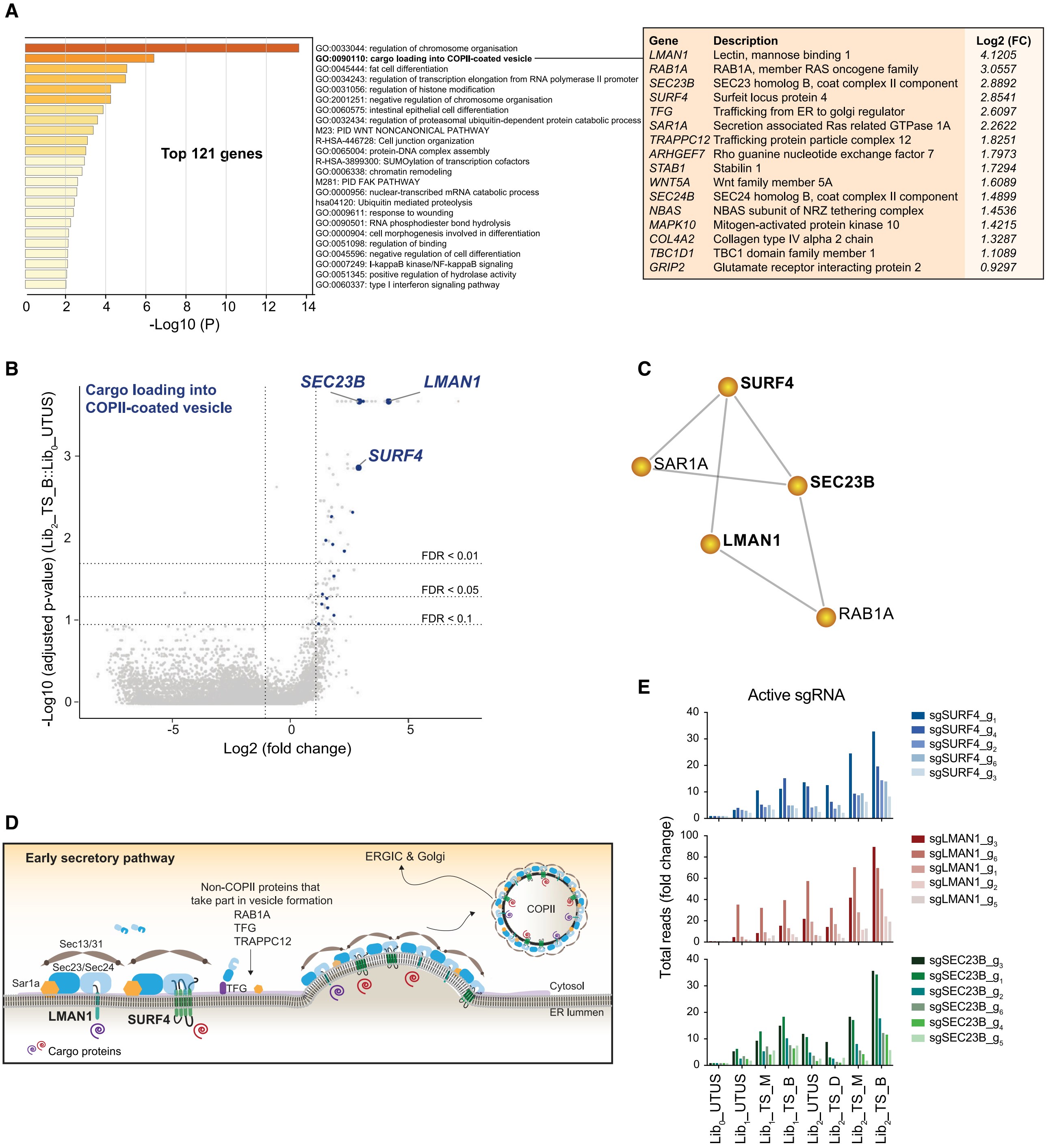

Figure 2. sgRNAs targeting genes encoding components of the early secretory pathway are enriched in cells with elevated intracellular α1AT polymers

(A) Gene Ontology (GO) enrichment analysis of the top 121 hits identified in the CRISPR screen and annotation of the 16 genes included in the GO term "cargo loading into COPII-coated vesicle" indicating the corresponding Log_2 (fold change) value for each gene.

(B) Volcano plot showing the Log_2 (fold change) and the Log_{10} (adjusted p value) of the genes targeted by sgRNAs in "treated and sorted" (TS) cells transduced with Lib_2 versus "untreated and unsorted" (UTUS) cells transduced with Lib_0. Genes above the horizontal dashed lines were significantly enriched in Lib_2. Genes of the GO term "cargo loading into COPII-coated vesicle" are in blue.

(C) Protein-protein interaction network (Metascape) of the 16 proteins encoded by the genes of the "cargo loading into COPII-coated vesicle" cluster.

(D) Cartoon of the early secretory pathway where relevant factors identified in the screen are depicted.

(E) Total reads for each active sgRNA targeting the selected genes for validation.

protein interaction network analysis of the proteins encoded by the 16 identified genes revealed that 5 of them form an independent network, highlighting their interconnectivity (Figure 2C). Thus, this screen hints at an important role for the early secretory pathway in specifying intracellular levels of α1AT polymers (Figure 2D). This finding was further supported by the observation that CHO-K1 Tet-on_α1AT^{H334D} cells treated with brefeldin A and FLI-06, two blockers of vesicular transport between the ER and the Golgi apparatus (Lippincott-Schwartz et al., 1989; Yonemura et al., 2016), reported increased intracellular levels of polymers over several hours of treatment (Figure S5).

Elevated intracellular α1AT^{H334D} polymer levels in cells lacking SURF4, LMAN1, and SEC23B

Of the genes targeted by guides enriched in the ‘‘brightest’’ cells, we deemed those encoding proteins with an ER luminal domain that could interact with polymers to be of particular interest. LMAN1 and SURF4, two transmembrane cargo receptors (Hauri et al., 2000; Reeves and Fried, 1995), satisfied that criterion. Another highly enriched gene, *SEC23B*, encoding the cytosolic component of the COPII machinery (Jensen and Schekman, 2011), was included as a reference (Figures 2A and 2B). Five of the six sgRNAs targeting each of these three genes were significantly enriched in the ‘‘brightest’’ population, adding confidence that they represent reliable hits (Figure 2E).

To validate the genotype-phenotype relationship suggested by the screen, we re-targeted *SURF4*, *LMAN1*, and *SEC23B* by CRISPR-Cas9-mediated gene disruption in parental CHO-K1 Tet-on_α1AT^{H334D} cells, using two guides mapping to separate exons (Figure 3A). Cells expressing wild-type (WT) α1AT (Ordóñez et al., 2013) were also targeted. Clonal knockout derivative cell lines were validated by genomic sequencing and, in the case of *SURF4* and *LMAN1*, by evidence for depletion of the proteins by immunoblotting (Figures 3B and 3C).

Disruption of *SURF4*, *LMAN1*, and *SEC23B* increased intracellular polymer levels as assessed by flow cytometry after immunostaining of polymeric α1AT^{H334D} (Figure 3D). These observations were confirmed by ELISA with two different antibodies: the polymer-specific Mab2C1 and a monoclonal antibody that recognizes all α1AT conformers (Mab3C11) (Figure 3E).

SURF4 and *LMAN1*, confirmed above as genes whose inactivation enhances levels of intracellular polymeric α1AT^{H334D}, play a broad role in trafficking of cargo out of the ER. Perturbations in ER function caused by protein misfolding or by impeded egress of proteins from the ER lead to ER stress and trigger the unfolded protein response (UPR), a protective and adaptive response aimed to re-establish ER homeostasis (Walter and Ron, 2011). Notably, *in vitro* studies indicate that brefeldin A, an inhibitor of protein transport from the ER to the Golgi apparatus, leads to the activation of the UPR (Citterio et al., 2008). Therefore, to gauge the contribution of any general perturbation to ER function that may arise from the inactivation of such genes, we turned to CHO-K1 S21 cells bearing *CHOP::GFP* and *XBP1s::Turquoise* UPR reporters (Sekine et al., 2016). *SURF4* and *LMAN1* were inactivated by sgRNA whose expression was linked to a mCherry reporter. This enabled scoring UPR activation in populations of mutant cells, free of the bias that might otherwise be introduced by clonal selection. No induction of the UPR reporters was observed following single *SURF4* and *LMAN1* inactivation. Inactivation of *HSPA5*, encoding the ER chaperone BiP, a positive control, strongly induced both UPR branches (Figures 4A and 4B). Furthermore, inactivation of *SURF4* in *LMAN1*$^{\Delta}$ CHO-K1 Tet-on_α1AT^{H334D} cells, bearing a *CHOP::GFP* reporter, did not induce the PERK (protein kinase RNA-like endoplasmic reticulum kinase) branch of the UPR (Figures 4C and 4D). However, global disruption of vesicular transport between the ER and the Golgi by treating cells with brefeldin A and FLI-06 strongly induced both UPR branches (Figure 4E). These observations indicate that inactivation of *SURF4* and *LMAN1* does not globally perturb ER protein homeostasis and suggests that the observed increase in polymers may arise from compromise in their roles as cargo receptors for polymerogenic α1AT.

LMAN1 and SURF4 promote trafficking of α1AT in CHO-K1 cells

LMAN1 has been previously implicated in mediating ER exit of WT monomeric α1AT (Nyfeler et al., 2008; Zhang et al., 2011). SURF4, by contrast, has been reported to lack such a function, at least in HEK293 cells (Emmer et al., 2018). To examine the roles of SURF4 and LMAN1 in the trafficking of polymerogenic α1AT^{H334D}, we performed pulse-chase experiments to compare the kinetics of α1AT secretion and the accumulation of polymers in parental *SURF4*$^{\Delta}$ and *LMAN1*$^{\Delta}$ CHO-K1 Tet-on_α1AT^{H334D} cells. Cells were pre-treated with a low concentration of doxycycline followed by radioactive pulse labeling for 20 min and a subsequent chase (Figure 5A). α1AT immunoprecipitation from cell lysates and culture media was performed with antibodies reactive with all forms of α1AT (total) or selective for polymers (Mab2C1) (Figure 5B). α1AT contains three N-glycosylation sites. Thus, the ER-associated 52-kDa α1AT^{H334D} species gradually appeared in the culture media as mature glycosylated species of 55 kDa (Figure 5B). Disruption of *LMAN1*, and to a lesser degree *SURF4*, led to a significant defect in the clearance of the ER form and appearance of the mature glycosylated form in the culture media (Figures 5B and 5C). This trend was even more conspicuous in terms of α1AT^{H334D} polymer secretion because *LMAN1*$^{\Delta}$ and *SURF4*$^{\Delta}$ cells accumulated more intracellular polymers than parental cells (Figures 5B and 5D). Similar findings were observed in an independently derived *SURF4*$^{\Delta}$ clone (Figure S6). Interestingly, both *LMAN1*$^{\Delta}$ and *SURF4*$^{\Delta}$ cells secreted proportionally fewer α1AT^{H334D} polymers than parental cells (Figures 5B and 5E).

Having confirmed a role for LMAN1 and SURF4 in trafficking of α1AT^{H334D}, we then sought to determine their role in trafficking of α1AT^{WT} in CHO cells. The same pulse-chase labeling procedure described above was applied to parental *SURF4*$^{\Delta}$ and *LMAN1*$^{\Delta}$ CHO-K1 Tet-on_α1AT^{WT} cells. Clearance of WT, monomeric α1AT from the ER was significantly delayed in *LMAN1*$^{\Delta}$ cells, consistent with previous observations (Nyfeler et al., 2008; Zhang et al., 2011), but also in *SURF4*$^{\Delta}$ cells, albeit to a lesser degree (Figures 5F and 5G). Of note, the accumulation of WT monomer in *SURF4*$^{\Delta}$ and *LMAN1*$^{\Delta}$ cells did not result in detectable polymer formation by ELISA.

These observations implicate both LMAN1 and SURF4 in trafficking of WT and polymerogenic α1AT in CHO-K1 cells. This explains enhanced intracellular accumulation of α1AT polymers

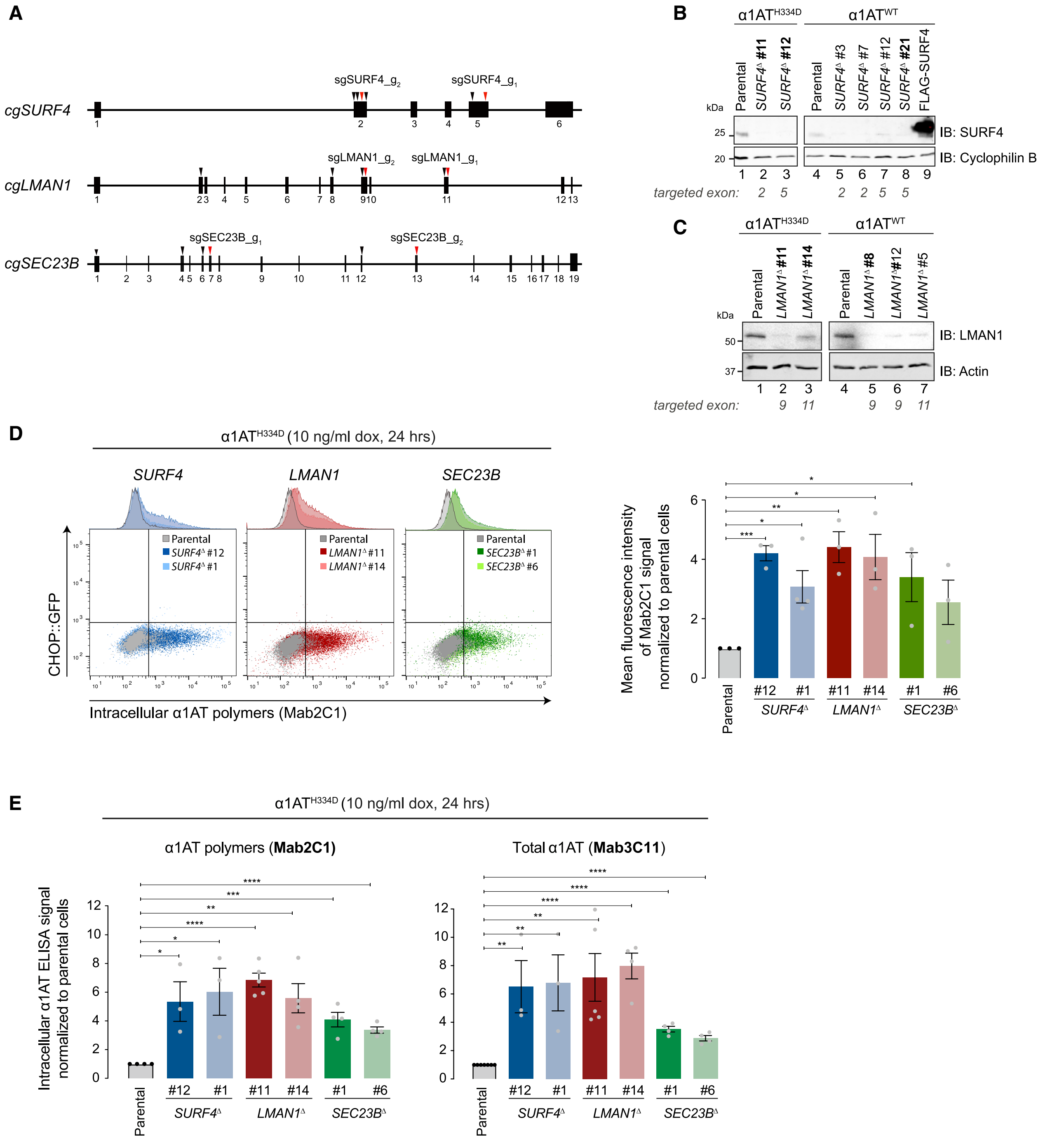

Figure 3. Disruption of *SURF4*, *LMAN1*, and *SEC23B* increases the intracellular levels of α1AT polymers in CHO-K1 cells

(A) Diagrams of the *Cricetulus griseus SURF4*, *LMAN1*, and *SEC23B* loci showing the target sites of the six sgRNAs (arrowheads) included in the CRISPR-Cas9 library. Red arrowheads indicate sgRNAs selected for validation.

(B and C) Immunoblots of SURF4 (B) and LMAN1 (C) in lysates of parental CHO-K1 Tet-on cells expressing either α1AT^{H334D} or α1AT^{WT} and several *SURF4*- and *LMAN1*-deleted derivatives. Clones selected for functional experiments are in boldface. Lysate of parental cells transfected with a FLAG-SURF4-ecoding plasmid served as a positive control.

(D) Dual-channel flow cytometry of intracellular levels of α1AT polymers and *CHOP::GFP* in CHO-K1 parental Tet-on_α1AT^{H334D} cells and two independent clones where *SURF4*, *LMAN1*, or *SEC23B* was disrupted. The bar graph shows the mean ± SEM of the Mab2C1-signal normalized to dox-treated parental cells from three or four independent experiments.

(E) As in (D), but plotting the intracellular α1AT signal from sandwich ELISA assays using the anti-polymer Mab2C1 (left panel) and the anti-total α1AT Mab3C11 (right panel). *p < 0.05, **p < 0.01, ***p < 0.001, ****p < 0.0001, unpaired t test.

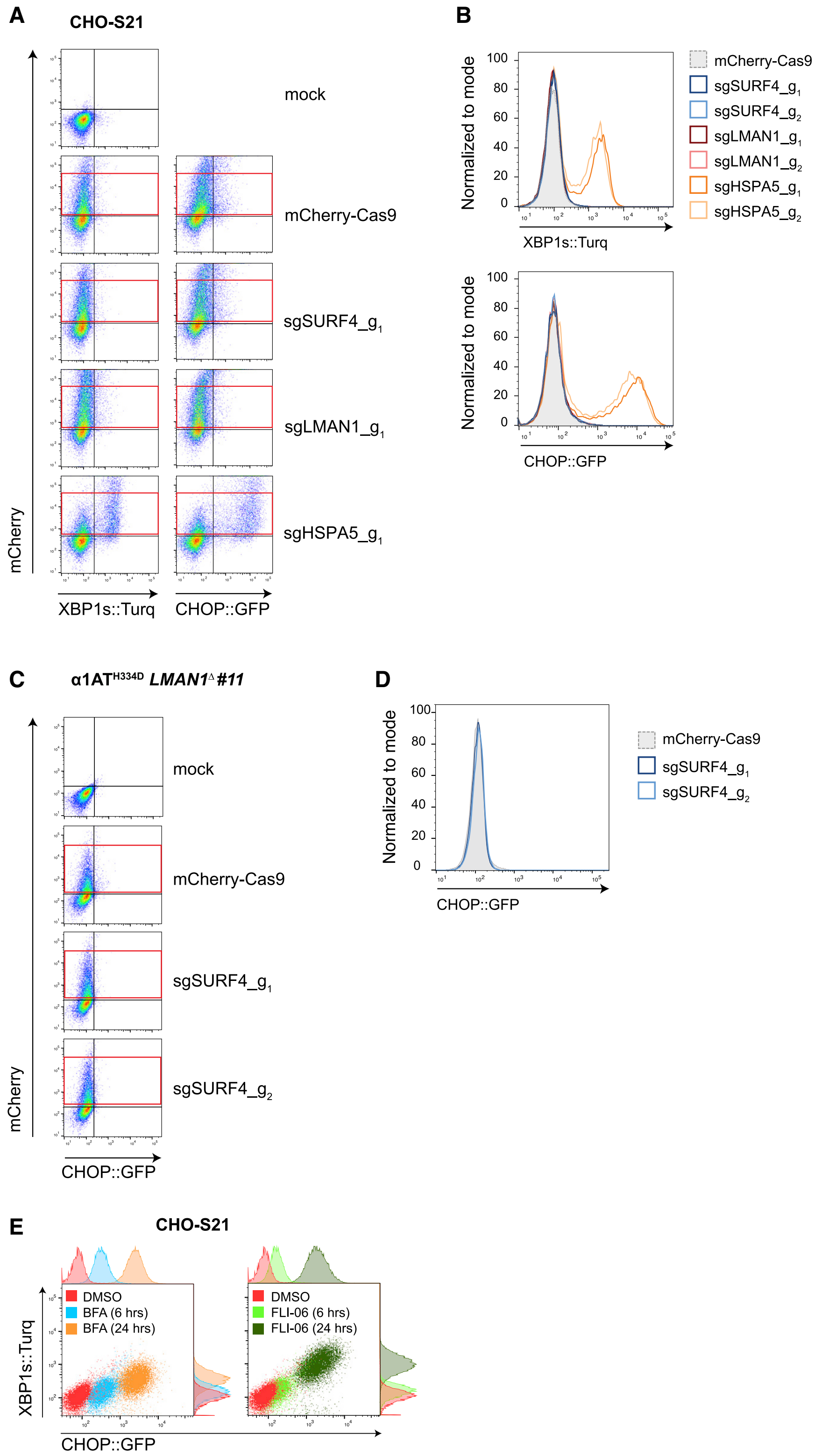

Figure 4. SURF4 and LMAN1 depletion does not activate the unfolded protein response

(A) Dual-channel flow cytometry of *XBP1s::Turquoise* or *CHOP::GFP* and mCherry signal in CHO-K1 S21 cells transiently transfected for 96 h with sgRNA-mCherry-Cas9 plasmids targeting *SURF4*, *LMAN1*, and *HSPA5* (BiP protein). Dot plots are representative of one experiment. The red rectangles delineate cells expressing moderate levels of mCherry-tagged plasmid selected for the histogram shown in (B).

(B) Distribution of the *XBP1s::Turquoise* and *CHOP::GFP* signals, in mCherry-positive cells gated by red rectangles in (A). The same experiment was repeated with equal results using a second sgRNA for each gene.

(C) Dual-channel flow cytometry of *CHOP::GFP* and mCherry signal in CHO-K1 Tet-on_α1AT^{H334D}_*LMAN1*$^{\Delta}$ cells transiently transfected for 96 h with two sgRNA-mCherry-Cas9 plasmids targeting *SURF4*. Dot plots are representative of one experiment. The red rectangles delineate cells expressing moderate levels of mCherry-tagged plasmid selected for the histogram shown in (D).

(D) Distribution of the *CHOP::GFP* signal, in mCherry-positive cells gated by red rectangles in (C).

(E) Dual-channel flow cytometry of *XBP1s::Turquoise* and *CHOP::GFP* in CHO-K1 S21 cells treated with two protein transport inhibitors: brefeldin A (BFA) and FLI-06. Treatments lasted 6 and 24 h. n = 1.

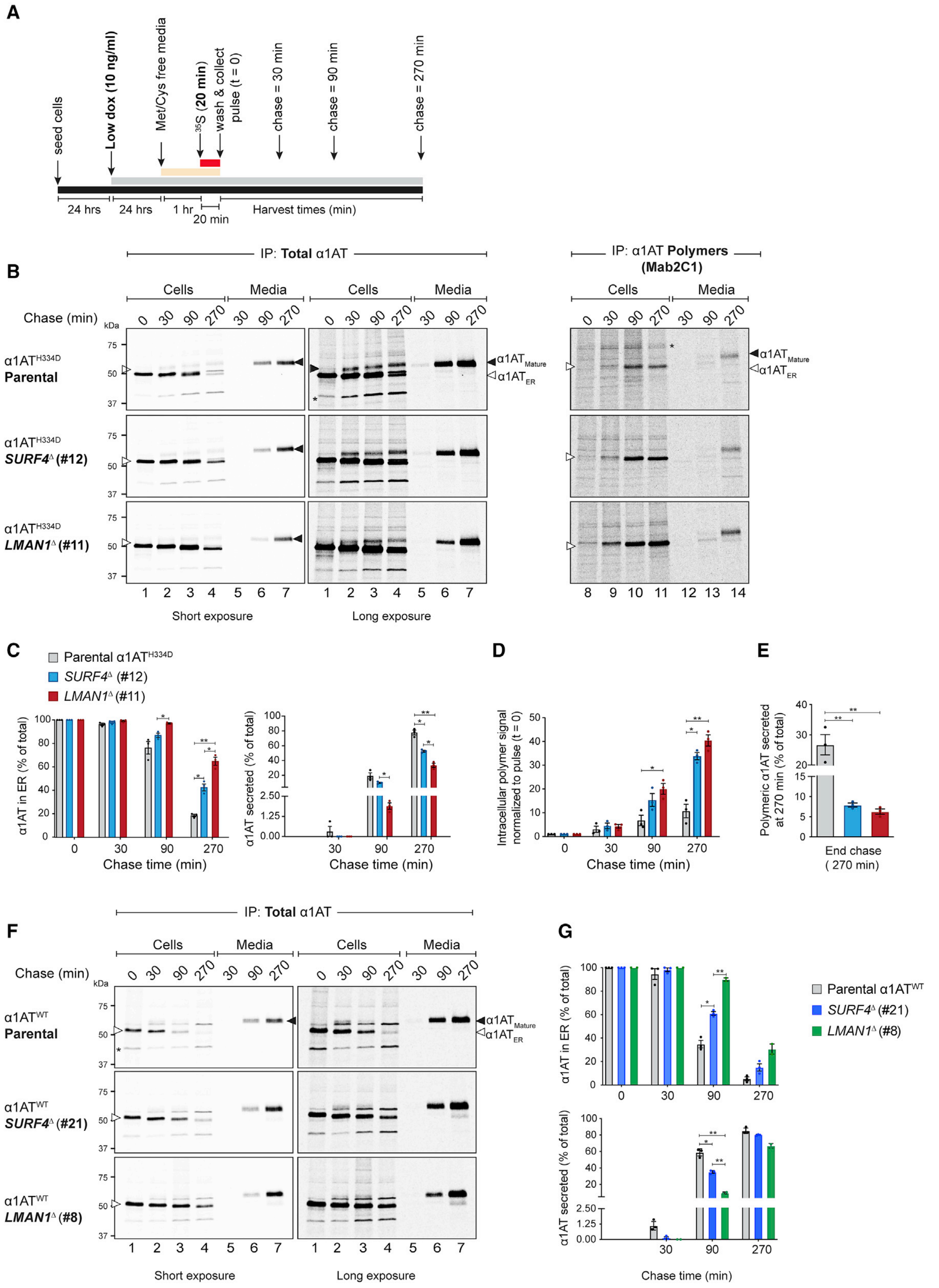

(legend on next page)

observed in the α1AT^{H334D}-expressing cells lacking either LMAN1 or SURF4.

SURF4 disruption preferentially impairs intracellular trafficking of α1AT polymers

SURF4 has been proposed as an ER cargo receptor that prioritizes export of large, polymeric proteins (Saegusa et al., 2018; Yin et al., 2018). This, together with our observations noted above, suggested the possibility that SURF4 might also have a role in facilitating the exit of α1AT polymers from the ER. To address this question, we modified the pulse-chase procedure: synthesis of α1AT^{H334D} was increased by treating the cells with a higher concentration of doxycycline, thus shifting the equilibrium toward polymer formation. Crucially, the pulse and chase windows were prolonged to allow clearance of the fast-trafficking (labeled) mutant monomeric species and thereby focused the analysis on the remaining polymers (Figure 6A).

The efficacy of these modifications is reflected in the appearance of a detectable pool of intracellular polymers at the end of the pulse and their persistence throughout the lengthy chase period, more conspicuously so in the *SURF4*$^{\Delta}$ and *LMAN1*$^{\Delta}$ cells (Figure 6B). In all three genotypes, labeled polymers also appeared in the culture media (Figure 6B), and these exhibited slower mobility on SDS-PAGE, compared with the cell-associated polymers. This observation is consistent with post-ER glycan modifications and indicates conventional trafficking through the secretory pathway.

In all three genotypes, intracellular polymer levels continued to increase after the pulse with levels peaking between 2.15 and 4.5 h chase (Figure 6C, upper panel). Thus, considering this peak as a reference point by which to track the fate of ER-localized polymers, we found that *SURF4*$^{\Delta}$ cells retained proportionally more polymers compared with parental or *LMAN1*$^{\Delta}$ cells (Figure 6C, lower panel). This finding correlated with a higher degree of co-localization of the polymers with the ER marker BiP in *SURF4*$^{\Delta}$ cells (Figures 6D and S7B). Notably, the kinetics of the ratio of secreted polymers to cell-associated polymers was significantly slower in *LMAN1*$^{\Delta}$ and *SURF4*$^{\Delta}$ cells (Figure 6E). Similar results were obtained with another independently derived *SURF4*$^{\Delta}$ clone (Figure S7).

These findings implicate both LMAN1 and SURF4 in secretion of α1AT polymers in CHO-K1 cells and suggest a preference of SURF4 for the transport of intracellular α1AT polymers out of the ER compared with LMAN1.

SURF4 interacts with α1AT in CHO-K1 cells

The interaction of LMAN1 and α1AT has been previously explored (Nyfeler et al., 2008). To assess possible physical interactions of SURF4 and α1AT, we transfected cells expressing α1AT^{H334D} or α1AT^{WT} with FLAG-tagged SURF4 and subjected them to crosslinking. FLAG-tagged SURF4 was selectively recovered by anti-FLAG immunoprecipitation, accompanied by either α1AT^{WT} or α1AT^{H334D} (Figures 7A, 7B, and 7E). Transfection with a 7xHis-tagged SURF4 provided an opportunity to recover SURF4-α1AT complexes under denaturing conditions, which also allowed more stringent wash steps. Nickel affinity pulldowns indicated that both α1AT^{WT} and α1AT^{H334D} were recovered in complex with 7xHis-SURF4 (Figures 7C–7E). Their recovery under denaturing conditions is consistent with a proximal interaction between the two species, although bridging by a third factor cannot be excluded.

The evidence provided here for an interaction between SURF4 and α1AT is in keeping with SURF4's functional role in trafficking of both polymeric and monomeric forms of α1AT.

DISCUSSION

By interfering with secretion, intracellular polymerization of mutant α1AT limits its plasma concentration and contributes to the loss-of-function features of α1AT deficiency. Simultaneously, polymer retention contributes to gain-of-function features, such as liver cirrhosis, while extracellular polymers appear to play a pro-inflammatory role in the lung (Lomas and Mahadeva, 2002) and elsewhere (Gross et al., 2009; Morris et al., 2011). Here, an unbiased genome-wide screen identified modifiers of intracellular levels of α1AT polymers, uncovering a previously under-appreciated role for cargo receptors in their active export from the ER and ultimately secretion of a fraction of the intracellular pool.

The strongest coherent signature to emerge from our screen was factors involved in cargo exit from the ER. These included LMAN1, a transmembrane cargo receptor known to have a role in the ER export of WT α1AT (Nyfeler et al., 2008; Zhang et al., 2011), validating the experimental approach. The screen

Figure 5. Altered intracellular trafficking of α1AT in *SURF4*- and *LMAN1*-disrupted cells

(A) Schema of the experimental design. Note the induction of α1AT expression with low concentration (10 ng/mL) of dox, ^{35}S-methionine/cysteine (Met/Cys) pulse labeling (20 min), and chase times (30–270 min).

(B) Short and long exposures of autoradiographs of SDS-PAGE gels load with labeled α1AT immunoprecipitated with a polyclonal antibody reactive with all forms of α1AT (left panels) or Mab2C1, selective for α1AT polymers (right panel) from lysates of parental CHO-K1 Tet-on_α1AT^{H334D} cells and their *SURF4*$^{\Delta}$ and *LMAN1*$^{\Delta}$ derivatives ("Cells") or the culture supernatant ("Media"). White arrowheads indicate the ER-associated form (α1AT$_{ER}$), and black arrowheads the mature-glycosylated form (α1AT$_{Mature}$). Asterisks (*) represent unspecific bands.

(C) Percentage of α1AT^{H334D} retained in the ER (α1AT$_{ER}$ in B, left panel) or secreted into the media (right panel) of total protein ("cell" signal + "media" signal) at each time point.

(D) Intracellular polymer signal normalized to α1AT polymer signal at pulse end (lane 8).

(E) Percentage of α1AT polymers present in the media of total protein at 270 min, calculated as in (C).

(F) As in (B), but using parental CHO-K1 Tet-on_α1AT^{WT} cells and their *SURF4*$^{\Delta}$ and *LMAN1*$^{\Delta}$ derivatives. Total α1AT from cells and media was immunoprecipitated as in (B).

(G) Percentage of α1AT^{WT} retained in the ER (upper panel) or secreted into the media (lower panel), calculated as in (C). Autoradiographs are representative of three independent experiments except for *LMAN1*$^{\Delta}$ (clone 8, n = 2).

Quantitative plots show the mean ± SEM. *$p < 0.05$, **$p < 0.01$. Two-way (C, D, and G) or one-way ANOVA (E) followed by Tukey's post hoc multiple comparison test.

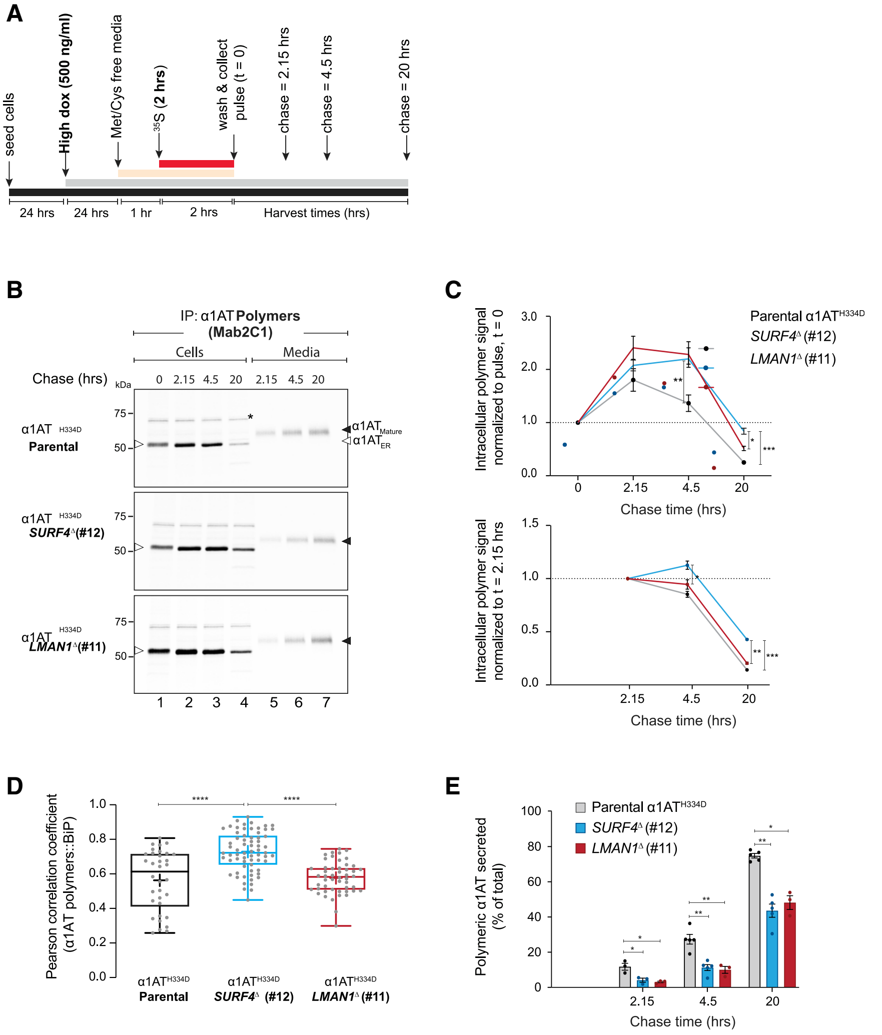

Figure 6. SURF4 and LMAN1 favor ER exit of α1AT polymers

(A) Schema of the experimental design. Note the induction of α1AT expression with a high concentration (500 ng/mL) of dox and the lengthy ^{35}S-Met/Cys pulse labeling period (2 h) and chase times (2.15–20 h).

(B) Autoradiographs of SDS-PAGE gels loaded with labeled α1AT immunoprecipitated with polymer-selective Mab2C1 from lysates of parental CHO-K1 Tet-on_α1AT^{H334D} cells and their *SURF4*$^{\Delta}$ and *LMAN1*$^{\Delta}$ derivatives (“Cells”) or the culture supernatant (“Media”). White arrowheads indicate the ER-associated form (α1AT$_{ER}$) and black arrowheads the mature-glycosylated form (α1AT$_{Mature}$). Asterisks (*) represent unspecific bands.

(C) Plot of the cell-associated α1AT polymer signal at the indicated times, normalized to the signal at pulse end (lane 1; upper panel) or to the signal at 2.15 h (lane 2; bottom panel).

(D) Pearson coefficient for the co-localization of α1AT polymers (Mab2C1-stained) with the ER marker BiP in dox-induced parental CHO-K1 Tet-on_α1AT^{H334D} cells (n = 34) and their *SURF4*$^{\Delta}$ (n = 67) and *LMAN1*$^{\Delta}$ (n = 50) derivatives (Figure S7B).

(E) Percentage of α1AT polymers present in the media of total protein (“cell” signal + “media” signal) at each time point in (B).

Quantitative plots show the mean ± SEM (n = 3–5). *p < 0.05, **p < 0.01, ***p < 0.001, ***p < 0.0001. Two-way (C and E) or one-way ANOVA (D) followed by Tukey’s post hoc multiple comparison test.

also implicated SURF4 in affecting the intracellular levels of α1AT polymers. SURF4, the human ortholog of the yeast cargo receptor Erv29p (Belden and Barlowe, 2001), has been shown to be a versatile multi-spanning cargo receptor that facilitates export of large proteins, such as the 550-kDa apolipoprotein B (Saegusa et al., 2018), small proteins, such as the 75-kDa PCSK9 (proprotein convertase subtilisin kexin 9) (Emmer et al., 2018), and soluble cargos that tend to aggregate within the ER (Yin et al., 2018). SURF4 has not been previously recognized to have a role in the trafficking of α1AT, but it has been reported to form multiprotein complexes with LMAN1, along with other components of the ER exit complex (Mitrovic et al., 2008). Therefore, we focused our attention on the mechanisms by which loss of these cargo receptors altered the intracellular fate of α1AT. These studies were carried out in genetically malleable CHO-K1 cells that recapitulate both ER morphology changes observed in hepatocytes of α1AT-deficient patients and the impairment of intracellular protein mobility observed in induced pluripotent stem cell-derived α1AT deficiency hepatocytes, confirming the utility of CHO-K1 cells as a discovery system for aspects of hepatocyte cellular physiology (Ordóñez et al., 2013; Segeritz et al., 2018). The screen was performed in cells expressing the highly polymerogenic King’s variant, that although not the most common α1AT mutant, recapitulates the phenotype observed in cells expressing the most common Z-α1AT variant and results in polymers that share the same structure, supporting the use of King’s mutant as a representative and comparable polymerogenic model of α1AT deficiency disease (Miranda et al., 2010; Ordóñez et al., 2013).

Disruption of either *LMAN1* or *SURF4* delayed trafficking of both polymerogenic α1AT^{H334D} and α1AT^{WT} out of the ER in this CHO-K1 system. Because polymerization is a concentration-dependent process (Lomas et al., 1993), impaired ER egress of mutant α1AT monomers could account for all the increase in intracellular polymer signal observed in the *LMAN1*$^{\Delta}$ and *SURF4*$^{\Delta}$ cells. This finding nonetheless emphasizes the fact that variation in the efficiency of monomer trafficking out of the ER could contribute to the clinical heterogeneity in polymer-induced liver disease (Wu et al., 1994).

Less anticipated were findings pointing to a role for LMAN1 and SURF4 in the egress of polymers out of the ER and, ultimately, in their secretion from cells. This insight was gleaned from cells expressing high levels of mutant α1AT^{H334D}, conditions predicted to shift the equilibrium in the ER toward

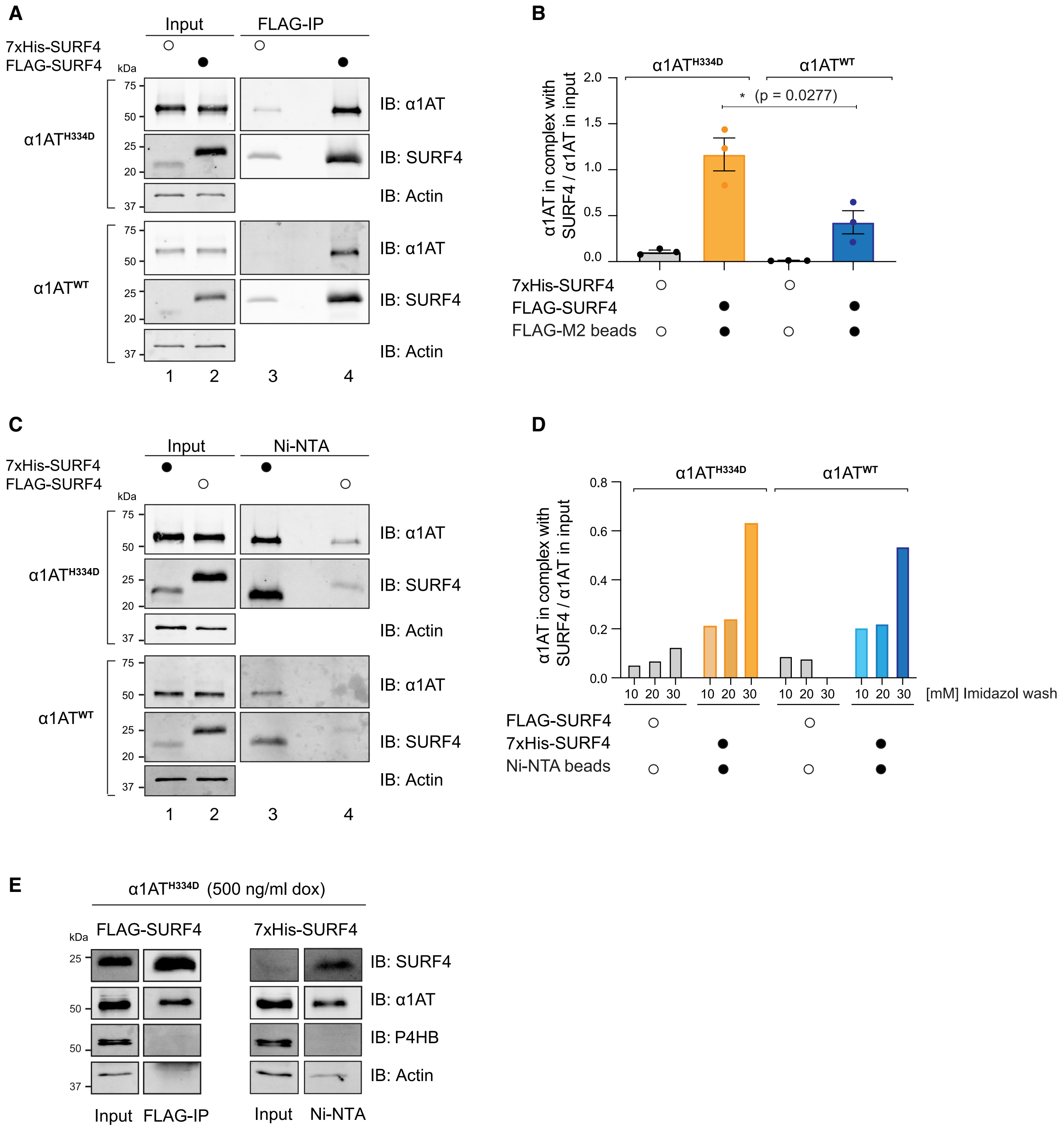

Figure 7. SURF4 interacts with α1AT

(A) Representative immunoblots of α1AT recovered in complex with FLAG-SURF4 (FLAG-IP [immunoprecipitation]) from CHO-K1 Tet-on cells expressing $\alpha1AT^{WT}$ or $\alpha1AT^{H334D}$ transfected with FLAG-tagged or 7xHis-tagged (as control) SURF4 plasmids and subjected to crosslinking.

(B) Ratio of the signal from the α1AT recovered in complex with FLAG-SURF4 to the α1AT signal in the "input." Shown is mean ± SEM from three independent experiments as in (A) (Student's t test).

(C) As in (A), but performing Ni-NTA affinity pulldowns under denaturing conditions on the same lysates used in (A). An imidazole gradient from 10 to 30 mM in the wash buffer was used across three experiments. This SDS-PAGE gel represents samples washed with 30 mM imidazole. Cells transfected with a FLAG-tagged SURF4 reported on the background (as control) in this assay.

(D) Ratio of the signal from the α1AT recovered in complex with 7xHis-tagged SURF4 to the α1AT signal in the "input" from three different experiments performed as in (C) in buffers with the indicated concentration of imidazole.

(E) Immunoprecipitations as in (A) and (C), including an antibody directed against P4HB (protein disulfide isomerase [PDI]), an abundant reference luminal ER-localized protein, reporting on the specificity of the interaction of SURF4 with α1AT. n = 2.

polymerization. Introducing a delay in the pulse-chase experiment that favored clearance of residual fast-trafficking labeled monomers focused the analysis on the fate of polymers. *LMAN1*$^{-\Delta}$ and even more so *SURF4*$^{\Delta}$ cells retained relatively more polymers and secreted relatively fewer polymers than parental cells. Co-localization of the excess polymers with the ER marker BiP was particularly conspicuous in the *SURF4*$^{\Delta}$ cells, supporting the idea that SURF4 may have an important role in clearing the ER of α1AT polymers and possibly other large cargos, as suggested previously (Saegusa et al., 2018).

Co-immunoprecipitation experiments hinted at direct contact, or at least close proximity, between SURF4 and α1AT. This was observed despite the absence from α1AT of an N-terminal motif previously reported to promote cargo binding to SURF4 (Yin et al., 2018) but also absent from other putative SURF4 cargos (e.g., PCSK9 and apolipoprotein B). Thus, at present, the basis for SURF4's ability to select monomeric and polymeric α1AT for export from the ER remains unknown.

The mobility of α1AT during SDS-PAGE suggests that polymeric α1AT found in the culture supernatant had undergone post-ER glycan modifications. This finding, together with the genetic evidence of a role for ER cargo receptors in its itinerary, suggests that at least a fraction of extracellular polymers found their way through the conventional secretory pathway. The existence of a pathway(s) by which misfolded ER proteins traffic out of the compartment, ultimately to be degraded in the lysosome (Fregno et al., 2018) (or out of the cell by extracellular vesicles as recently reported for the Z α1AT variant; Khodayari et al., 2019) raises the possibility that LMAN1 or SURF4 also restrain intracellular polymer levels by promoting a trafficking event that contributes to their intracellular degradation. These issues remain unsettled even in our CHO-K1 model. Nonetheless, the role of ER cargo receptors in the itinerary of α1AT monomers and polymers highlighted in this study conjures the possibility of mechanism-based interventions to alter the balance of polymers retained in cells, degraded intracellularly, or secreted and could represent new therapeutic targets for the underlying lung disease.

STAR★METHODS

Detailed methods are provided in the online version of this paper and include the following:

- KEY RESOURCES TABLE
- RESOURCE AVAILABILITY
  - Lead contact
  - Materials availability
  - Data and code availability
- EXPERIMENTAL MODEL AND SUBJECT DETAILS
  - CHO-K1-derived adherent cells
  - HEK293T-derived adherent cell lines
- METHOD DETAILS
  - Lentiviral production
  - Intracellular polymer staining for FACS screen and flow cytometry
  - Whole genome CRISPR screen
  - Validation of candidate genes
  - Mammalian cell lysates, sandwich ELISA, and immunoblotting
  - [^{35}S] metabolic labeling and immunoprecipitation
  - Cross-linking and co-immunoprecipitation
  - Confocal microscopy
- QUANTIFICATION AND STATISTICAL ANALYSIS

SUPPLEMENTAL INFORMATION

Supplemental information can be found online at https://doi.org/10.1016/j.celrep.2021.109144.

ACKNOWLEDGMENTS

We thank the CIMR flow cytometry (Reiner Schulte, Chiara Cossetti, and Gabriela Grondys-Kotarba) and microscopy teams (Matthew Gratian and Mark Bowen) for technical support; Marcella Ma and Brian Lam (CRUK) for assistance with next-generation sequencing (NGS); Peter Sterk (University of Cambridge) for NGS data analysis support; and our lab members, especially Steffen Preissler (CIMR), for critical comments and advice. This study was supported in whole by a research grant from Wellcome (200848/Z/16/Z).

AUTHOR CONTRIBUTIONS

A.O. conceived, initiated, and led the project; designed and conducted the experiments; analyzed and interpreted the data; prepared figures and tables; and wrote the first draft of the manuscript. H.P.H. designed the CHO CRISPR-Cas9 library, contributed experimentally with the lentiviral library and in data analysis, and reviewed the manuscript. S.J.M. contributed to discussion and revision of the manuscript. D.R. conceived and oversaw the project, interpreted the data, and co-wrote the manuscript. All authors read and approved the final manuscript.

DECLARATION OF INTERESTS

The authors declare no competing interests.

Received: October 1, 2020
Revised: March 1, 2021
Accepted: April 26, 2021
Published: May 18, 2021

REFERENCES

Amin-Wetzel, N., Saunders, R.A., Kamphuis, M.J., Rato, C., Preissler, S., Harding, H.P., and Ron, D. (2017). A J-Protein co-chaperone recruits BiP to monomerize IRE1 and repress the unfolded protein response. Cell *171*, 1625–1637.e13.

Avezov, E., Cross, B.C.S., Kaminski Schierle, G.S., Winters, M., Harding, H.P., Melo, E.P., Kaminski, C.F., and Ron, D. (2013). Lifetime imaging of a fluorescent protein sensor reveals surprising stability of ER thiol redox. J. Cell Biol. *201*, 337–349.

Belden, W.J., and Barlowe, C. (2001). Role of Erv29p in collecting soluble secretory proteins into ER-derived transport vesicles. Science *294*, 1528–1531.

Carrell, R.W., and Lomas, D.A. (2002). Alpha1-antitrypsin deficiency—a model for conformational diseases. N. Engl. J. Med. *346*, 45–53.

Citterio, C., Vichi, A., Pacheco-Rodriguez, G., Aponte, A.M., Moss, J., and Vaughan, M. (2008). Unfolded protein response and cell death after depletion of brefeldin A-inhibited guanine nucleotide-exchange protein GBF1. Proc. Natl. Acad. Sci. USA *105*, 2877–2882.

D'Arcangelo, J.G., Stahmer, K.R., and Miller, E.A. (2013). Vesicle-mediated export from the ER: COPII coat function and regulation. Biochim. Biophys. Acta *1833*, 2464–2472.

Emmer, B.T., Hesketh, G.G., Kotnik, E., Tang, V.T., Lascuna, P.J., Xiang, J., Gingras, A.C., Chen, X.W., and Ginsburg, D. (2018). The cargo receptor SURF4 promotes the efficient cellular secretion of PCSK9. eLife *7*, e38839.

Eriksson, S., Carlson, J., and Velez, R. (1986). Risk of cirrhosis and primary liver cancer in alpha 1-antitrypsin deficiency. N. Engl. J. Med. *314*, 736–739.

Fra, A., Cosmi, F., Ordoñez, A., Berardelli, R., Perez, J., Guadagno, N.A., Corda, L., Marciniak, S.J., Lomas, D.A., and Miranda, E. (2016). Polymers of Z α1-antitrypsin are secreted in cell models of disease. Eur. Respir. J. *47*, 1005–1009.

Fregno, I., Fasana, E., Bergmann, T.J., Raimondi, A., Loi, M., Soldà, T., Galli, C., D'Antuono, R., Morone, D., Danieli, A., et al. (2018). ER-to-lysosome-associated degradation of proteasome-resistant ATZ polymers occurs via receptor-mediated vesicular transport. EMBO J. *37*, e99259.

Gomez-Navarro, N., and Miller, E. (2016). Protein sorting at the ER-Golgi interface. J. Cell Biol. *215*, 769–778.

Gooptu, B., and Lomas, D.A. (2008). Polymers and inflammation: disease mechanisms of the serpinopathies. J. Exp. Med. *205*, 1529–1534.

Gooptu, B., Dickens, J.A., and Lomas, D.A. (2014). The molecular and cellular pathology of α_1-antitrypsin deficiency. Trends Mol. Med. *20*, 116–127.

Gross, B., Grebe, M., Wencker, M., Stoller, J.K., Bjursten, L.M., and Janciauskiene, S. (2009). New Findings in PiZZ alpha1-antitrypsin deficiency-related panniculitis. Demonstration of skin polymers and high dosing requirements of intravenous augmentation therapy. Dermatology *218*, 370–375.

Harding, H.P., Ordonez, A., Allen, F., Parts, L., Inglis, A.J., Williams, R.L., and Ron, D. (2019). The ribosomal P-stalk couples amino acid starvation to GCN2 activation in mammalian cells. eLife *8*, e50149.

Hauri, H.P., Kappeler, F., Andersson, H., and Appenzeller, C. (2000). ERGIC-53 and traffic in the secretory pathway. J. Cell Sci. *113*, 587–596.

Hjelm, L.N., Chin, E.L., Hegde, M.R., Coffee, B.W., and Bean, L.J. (2010). A simple method to confirm and size deletion, duplication, and insertion mutations detected by sequence analysis. J. Mol. Diagn. *12*, 607–610.

Jensen, D., and Schekman, R. (2011). COPII-mediated vesicle formation at a glance. J. Cell Sci. *124*, 1–4.

Khodayari, N., Oshins, R., Alli, A.A., Tuna, K.M., Holliday, L.S., Krotova, K., and Brantly, M. (2019). Modulation of calreticulin expression reveals a novel exosome-mediated mechanism of Z variant α_1-antitrypsin disposal. J. Biol. Chem. *294*, 6240–6252.

Kroeger, H., Miranda, E., MacLeod, I., Pérez, J., Crowther, D.C., Marciniak, S.J., and Lomas, D.A. (2009). Endoplasmic reticulum-associated degradation (ERAD) and autophagy cooperate to degrade polymerogenic mutant serpins. J. Biol. Chem. *284*, 22793–22802.

Li, W., Xu, H., Xiao, T., Cong, L., Love, M.I., Zhang, F., Irizarry, R.A., Liu, J.S., Brown, M., and Liu, X.S. (2014). MAGeCK enables robust identification of essential genes from genome-scale CRISPR/Cas9 knockout screens. Genome Biol. *15*, 554.

Lippincott-Schwartz, J., Yuan, L.C., Bonifacino, J.S., and Klausner, R.D. (1989). Rapid redistribution of Golgi proteins into the ER in cells treated with brefeldin A: evidence for membrane cycling from Golgi to ER. Cell *56*, 801–813.

Lomas, D.A., and Mahadeva, R. (2002). Alpha1-antitrypsin polymerization and the serpinopathies: pathobiology and prospects for therapy. J. Clin. Invest. *110*, 1585–1590.

Lomas, D.A., Evans, D.L., Stone, S.R., Chang, W.S., and Carrell, R.W. (1993). Effect of the Z mutation on the physical and inhibitory properties of alpha 1-antitrypsin. Biochemistry *32*, 500–508.

Mahadeva, R., Atkinson, C., Li, Z., Stewart, S., Janciauskiene, S., Kelley, D.G., Parmar, J., Pitman, R., Shapiro, S.D., and Lomas, D.A. (2005). Polymers of Z alpha1-antitrypsin co-localize with neutrophils in emphysematous alveoli and are chemotactic in vivo. Am. J. Pathol. *166*, 377–386.

Miranda, E., Pérez, J., Ekeowa, U.I., Hadzic, N., Kalsheker, N., Gooptu, B., Portmann, B., Belorgey, D., Hill, M., Chambers, S., et al. (2010). A novel monoclonal antibody to characterize pathogenic polymers in liver disease associated with alpha1-antitrypsin deficiency. Hepatology *52*, 1078–1088.

Mitrovic, S., Ben-Tekaya, H., Koegler, E., Gruenberg, J., and Hauri, H.P. (2008). The cargo receptors Surf4, endoplasmic reticulum-Golgi intermediate compartment (ERGIC)-53, and p25 are required to maintain the architecture of ERGIC and Golgi. Mol. Biol. Cell *19*, 1976–1990.

Morris, H., Morgan, M.D., Wood, A.M., Smith, S.W., Ekeowa, U.I., Herrmann, K., Holle, J.U., Guillevin, L., Lomas, D.A., Perez, J., et al. (2011). ANCA-associated vasculitis is linked to carriage of the Z allele of α_1 antitrypsin and its polymers. Ann. Rheum. Dis. *70*, 1851–1856.

Morrison, H.M., Kramps, J.A., Burnett, D., and Stockley, R.A. (1987). Lung lavage fluid from patients with alpha 1-proteinase inhibitor deficiency or chronic obstructive bronchitis: anti-elastase function and cell profile. Clin. Sci. (Lond.) *72*, 373–381.

Mulgrew, A.T., Taggart, C.C., Lawless, M.W., Greene, C.M., Brantly, M.L., O'Neill, S.J., and McElvaney, N.G. (2004). Z alpha1-antitrypsin polymerizes in the lung and acts as a neutrophil chemoattractant. Chest *125*, 1952–1957.

Nyfeler, B., Reiterer, V., Wendeler, M.W., Stefan, E., Zhang, B., Michnick, S.W., and Hauri, H.P. (2008). Identification of ERGIC-53 as an intracellular transport receptor of alpha1-antitrypsin. J. Cell Biol. *180*, 705–712.

Ordóñez, A., Snapp, E.L., Tan, L., Miranda, E., Marciniak, S.J., and Lomas, D.A. (2013). Endoplasmic reticulum polymers impair luminal protein mobility and sensitize to cellular stress in alpha1-antitrypsin deficiency. Hepatology *57*, 2049–2060.

Ran, F.A., Hsu, P.D., Wright, J., Agarwala, V., Scott, D.A., and Zhang, F. (2013). Genome engineering using the CRISPR-Cas9 system. Nat. Protoc. *8*, 2281–2308.

Reeves, J.E., and Fried, M. (1995). The surf-4 gene encodes a novel 30 kDa integral membrane protein. Mol. Membr. Biol. *12*, 201–208.

Saegusa, K., Sato, M., Morooka, N., Hara, T., and Sato, K. (2018). SFT-4/Surf4 control ER export of soluble cargo proteins and participate in ER exit site organization. J. Cell Biol. *217*, 2073–2085.

Schindelin, J., Arganda-Carreras, I., Frise, E., Kaynig, V., Longair, M., Pietzsch, T., Preibisch, S., Rueden, C., Saalfeld, S., Schmid, B., et al. (2012). Fiji: an open-source platform for biological-image analysis. Nat. Methods *9*, 676–682.

Segeritz, C.P., Rashid, S.T., de Brito, M.C., Serra, M.P., Ordonez, A., Morell, C.M., Kaserman, J.E., Madrigal, P., Hannan, N.R.F., Gatto, L., et al. (2018). hiPSC hepatocyte model demonstrates the role of unfolded protein response and inflammatory networks in α_1-antitrypsin deficiency. J. Hepatol. *69*, 851–860.

Sekine, Y., Zyryanova, A., Crespillo-Casado, A., Amin-Wetzel, N., Harding, H.P., and Ron, D. (2016). Paradoxical Sensitivity to an Integrated Stress Response Blocking Mutation in Vanishing White Matter Cells. PLoS ONE *11*, e0166278.

Shalem, O., Sanjana, N.E., Hartenian, E., Shi, X., Scott, D.A., Mikkelson, T., Heckl, D., Ebert, B.L., Root, D.E., Doench, J.G., and Zhang, F. (2014). Genome-scale CRISPR-Cas9 knockout screening in human cells. Science *343*, 84–87.

Tan, L., Dickens, J.A., Demeo, D.L., Miranda, E., Perez, J., Rashid, S.T., Day, J., Ordoñez, A., Marciniak, S.J., Haq, I., et al. (2014). Circulating polymers in α1-antitrypsin deficiency. Eur. Respir. J. *43*, 1501–1504.

Tan, L., Perez, J., Mela, M., Miranda, E., Burling, K.A., Rouhani, F.N., DeMeo, D.L., Haq, I., Irving, J.A., Ordóñez, A., et al. (2015). Characterising the association of latency with α(1)-antitrypsin polymerisation using a novel monoclonal antibody. Int. J. Biochem. Cell Biol. *58*, 81–91.

Walter, P., and Ron, D. (2011). The unfolded protein response: from stress pathway to homeostatic regulation. Science *334*, 1081–1086.

Wu, Y., Whitman, I., Molmenti, E., Moore, K., Hippenmeyer, P., and Perlmutter, D.H. (1994). A lag in intracellular degradation of mutant alpha 1-antitrypsin correlates with the liver disease phenotype in homozygous PiZZ alpha 1-antitrypsin deficiency. Proc. Natl. Acad. Sci. USA *91*, 9014–9018.

Yin, Y., Garcia, M.R., Novak, A.J., Saunders, A.M., Ank, R.S., Nam, A.S., and Fisher, L.W. (2018). Surf4 (Erv29p) binds amino-terminal tripeptide motifs of soluble cargo proteins with different affinities, enabling prioritization of their exit from the endoplasmic reticulum. PLoS Biol. *16*, e2005140.

Yonemura, Y., Li, X., Müller, K., Krämer, A., Atigbire, P., Mentrup, T., Feuerhake, T., Kroll, T., Shomron, O., Nohl, R., et al. (2016). Inhibition of cargo export at ER exit sites and the trans-Golgi network by the secretion inhibitor FLI-06. J. Cell Sci. *129*, 3868–3877.

Zhang, B., Zheng, C., Zhu, M., Tao, J., Vasievich, M.P., Baines, A., Kim, J., Schekman, R., Kaufman, R.J., and Ginsburg, D. (2011). Mice deficient in LMAN1 exhibit FV and FVIII deficiencies and liver accumulation of α1-antitrypsin. Blood *118*, 3384–3391.

Zhou, Y., Zhou, B., Pache, L., Chang, M., Khodabakhshi, A.H., Tanaseichuk, O., Benner, C., and Chanda, S.K. (2019). Metascape provides a biologist-oriented resource for the analysis of systems-level datasets. Nat. Commun. *10*, 1523.

Zlatic, S.A., Ryder, P.V., Salazar, G., and Faundez, V. (2010). Isolation of labile multi-protein complexes by in vivo controlled cellular cross-linking and immuno-magnetic affinity chromatography. J. Vis. Exp. *2010*, 1855.

STAR★METHODS

KEY RESOURCES TABLE

REAGENT or RESOURCE	SOURCE	IDENTIFIER
Antibodies		
Monoclonal Mouse anti-α1AT polymer-specific 2C1	Miranda et al., 2010	PMID:20583215
Monoclonal Mouse anti-total α1AT 3C11	Tan et al., 2015	PMID:25462157
Polyclonal Rabbit anti-total α1AT	Agilent, Dako	RRID:AB_2335672
Polyclonal Rabbit anti-ERGIC-53	Sigma	RRID:AB_532237
Polyclonal Rabbit anti-SURF4	Invitrogen	RRID:AB_2689252
Monoclonal Mouse anti-FLAG M2	Sigma	RRID:AB_262044
Polyclonal Rabbit anti-cyclophilin B	Abcam	RRID:AB_443295
Monoclonal Mouse anti-actin	Abcam	RRID:AB_303668
Polyclonal Chicken anti-hamster BiP	Avezov et al., 2013	PMID:23589496
Monoclonal Mouse anti-PDI	Enzo Life Sciences	RRID:AB_10615355
Goat anti-Mouse IgG (H+L) Cross-Adsorbed Secondary Antibody, DyLight 633	Thermo Fisher Scientific	RRID:AB_1965952
Chemicals, peptides, and recombinant proteins		
Doxycycline	Sigma	Cat#D9861
DMEM	Sigma	Cat#D6429
Tet Free Serum	Pan-Biotech	Cat#P30-3602
HyClone II Serum	Thermo Fisher Scientific	Cat#SH30066.03
Penicillin/Streptomycin	Sigma	Cat#P0781
L-glutamine	Sigma	Cat#G7513
Non-essential amino acids solution	Sigma	Cat#M7145
Hygromycin B	Thermo Fisher Scientific	Cat#10687010
G-418	Melford	Cat#G0175
Nutrient Mixture F12	Sigma	Cat#N4888
Lipofectamine LTX	Thermo Fisher Scientific	Cat#A12621
TransIT-293 Transfection Reagent	Mirus	Cat#MIR2704
Bafilomycin A1	Sigma	Cat#B1793
Dithiobis(succinimidyl propionate) (DSP)	Thermo Scientific Pierce	Cat#22585
Puromycin	MERCK-milipore	Cat#540222
EDTA-free Protease inhibitor Cocktail	Roche	Cat#11873580001
DMEM (-Glu/-Met/-Cys)	GIBCO	Cat#21013024
Easy TagTM Express ^{35}S Protein Labeling Mix	Perkin-Elmer	NEG072007MC
Protein A-Sepharose	Sigma	Cat#P3391
Protein G- Sepharose 4B fast flow	Sigma	Cat#P3296
Anti-FLAG M2 Affinity Gel	Sigma	Cat#F3165
Ni-NTA Agarose beads	QIAGEN	Cat#30210
Brefeldin A, BFA	LC Laboratories	Cat#B-8500
FLI-06	Sigma	Cat#SML0975
Deposited data		
Raw and analyzed data. See table for analyzed data	This study	GSE158574
Processed high-throughput sequencing data, including the full gene-ranking list of top hits (Table S1).	This study	GSE158574

(Continued on next page)

Continued		
REAGENT or RESOURCE	SOURCE	IDENTIFIER
Experimental models: Cell lines		
Hamster: CHO Tet-on [α1AT^{H334D}_CHOP::GFP_Cas9]	This study	N/A
Hamster: CHO Tet-on [α1AT^{H334D}_CHOP::GFP]	This study	N/A
Hamster: CHO Tet-on [α1AT^{WT}_CHOP::GFP]	This study	N/A
Hamster: CHO Tet-on [α1AT^{H334D}]	Ordóñez et al., 2013	PMID: 23197448
Hamster: CHO Tet-on [α1AT^{W}]	Ordóñez et al., 2013	PMID: 23197448
Hamster: CHO-S21 dual reporter [CHOP::GFP; XBP1::Turquoise]	Sekine et al., 2016	PMID: 27812215
Human: HEK293T	ATCC	RRID:CVCL_0063
For full list see Table S2	This study	N/A
Oligonucleotides		
Oligo2182_sgRNA_outer_MluI_short_F (primer for PCR of pKLV CHO_CRISPR library for recloning in UK1789):CAGCAGAGATCCAGTTTGGTTAGTACC	This study	N/A
Oligo1432_ P5-sgRNA_inner_F (primer for barcoding and adapting lentiGuide PCR products from CRISPR library screening for NGS): AATGATACGGCGACCACCGAGATCTAC ACTCTCTTGTGGAAAGGACGAAACACCG	Harding et al., 2019	PMID: 31749445
For full list see Table S3	This study	N/A
Recombinant DNA		
UK1610_pSpCas9(BB)-2A-mCherry	Amin-Wetzel et al., 2017	PMID:29198525
UK1700_pMD2.G	Addgene	RRID:Addgene_12259
UK1701_psPAX2	Addgene	RRID:Addgene_12260
UK1702_LentiGuide-puro	Addgene	Plasmid#52963
UK1714_Lenti-Cas9	This study	N/A
UK1717_EGFPsgRNA_lentiGuide-Puro	This study	N/A
UK1789_pKLV-U6gRNA(BbsI)-PGKpuro2ABFP	Addgene	RRID:Addgene_50946
For full list see Table S4	This study	N/A
Software and algorithms		
MAGeCK	Li et al., 2014	PMID:25476604
Metascape	Zhou et al., 2019	PMCID:6447622
FlowJo	BD	https://www.flowjo.com/
Fiji (ImageJ 1.53c NIH)	Schindelin et al., 2012	https://imagej.nih.gov/ij/
Prism V8	GraphPad	N/A
Volocity V6.3	Perkin Elmer	N/A

RESOURCE AVAILABILITY

Lead contact

Further information and requests for resources and reagents should be directed to and will be fulfilled by the Lead Contact, Adriana Ordóñez (aog23@cam.ac.uk).

Materials availability

Plasmids and cell lines generated in this study are available upon written request to the Lead contact. Please consult the list of unique reagents in Tables S2–S4 and Key Resources Table.

Data and code availability

The raw and processed high-throughput sequencing data from the CRISPR screen reported in this study are available at NCBI's Gene Expression Omnibus (GEO, accession number: GSE158574). The processed data includes the full gene-ranking list of top hits.

EXPERIMENTAL MODEL AND SUBJECT DETAILS

CHO-K1-derived adherent cells

Chinese hamster ovarian epithelial cells expressing human α1AT^{WT} or the polymerogenic α1AT^{H334D} mutant under a tetracycline inducible promoter (Ordóñez et al., 2013) were maintained in DMEM (D6429, Sigma) supplemented with 10% Tet-free serum (Pan-Biotech), 1x Penicillin-Streptomycin (P0781, Sigma), 1x non-essential-amino-acids (M7145, Sigma), 2 mM L-glutamine (G7513, Sigma), 200 μg/mL G418 (G0175, Melford) and 500 μg/mL of Hygromycin B (10687010, Thermo) at 37°C and 5% CO_2. Depending on the experiment, α1AT expression was induced with 10 ng/ml (‘low dox’) or 500 ng/ml (‘high dox’) doxycycline for 24 hr. Although not relevant for these experiments, the open reading frame of *Cricetulus griseus DDIT3* locus was replaced by GFP (*CHOP::GFP* reporter) in the parental CHO-K1 Tet-on cells. For the CRISPR-Cas9 screen we stably introduced the Cas9 nuclease into CHO-K1 Tet-on_α1AT^{H334D} cells via lentiviral transduction (UK1714, see Tables S3 and S4). Cas9 activity in derivative cell lines was confirmed by targeting the *CHOP::GFP* reporter with a EGFP-targeting sgRNA (UK1717) followed by induction of ER stress.

CHO-K1 S21 cells bearing *CHOP::GFP* and *XBP1s::Turquoise* reporters (Sekine et al., 2016) were maintained in Nutrient Mixture F12 (N4888,Sigma) supplemented with 10% Fetal Calf serum (FetalClone II, Thermo), 1 × Penicillin-Streptomycin (P0781, Sigma) and 2 mM L-glutamine (G7513, Sigma) at 37°C and 5% CO_2. These cells were used in the experiments described in Figure 4.

HEK293T-derived adherent cell lines

Human embryonic kidney 293 cells T cells (ATCC CRL-3216) were maintained in DMEM supplemented as above. All cells were grown at 37°C and 5% CO_2.

Where indicated, cells were treated with 10 - 500 ng/ml doxycycline (dox), 10 μg/ml brefeldin A (BFA, B8500, LC laboratories) and 10 μM FLI-06 (SML0975, Sigma). All the cell lines generated in this study are indicated in Table S2 and Key Resources Table. All experiments were performed at cell densities of 70%–90% confluence.

METHOD DETAILS

Lentiviral production

Lentiviral particles were produced by transfecting HEK293T cells with the library plasmids (UK2561, UK2321 and UK2378) together with the packaging plasmids psPAX2 (UK1701) and pMD2.G (UK1700) at a 10:7.5:5 ratio using TransIT-293 Transfection Reagent (MIR2704, Mirus) according to the manufacturer’s instructions. The supernatant containing the viral particles was collected 48 hr after transfection, filtered through a 0.45 μm filter, and directly used to infect CHO-K1 cells seeded in 6-well plates for viral titration.

Intracellular polymer staining for FACS screen and flow cytometry

Cells were washed twice with PBS, collected in PBS containing 4 mM EDTA and 0.2% BSA and fixed in 1% formaldehyde for 10 min. Fixative was washed-out at 700 × *g* for 5 min and cells were permeabilized in blocking buffer [PBS containing 0.1% Triton X-100 and 10% FBS] for 20 min, incubated with the primary α1AT polymer-specific monoclonal antibody 2C1 (Mab2C1) (Miranda et al., 2010) for 30 min, washed three times in blocking solution, and then incubated with the secondary DyLight 633-labeled anti-mouse antibody for 20 min. Cells were washed, resuspended in PBS containing 2 mM EDTA and 2% FBS), filtered and sorted on an Influx cell sorter (BD) or analyzed by flow cytometry (20,000 cells/sample) using a LSRFortessa cell analyzer (BD). In order to reduce cell clumping, a cell density of $\sim 2 \times 10^6$ cells/ml was adjusted and all incubations were done with orbital agitation at room temperature or 4°C, when required. Cells were gated by forward (FSC-A) and side scatter (SSC-A) for live cells, then for single cells using FSC-A/FSC-H. α1AT polymers (Mab2C1 signal) were detected by excitation at 640 nm and monitoring emission at 670/14 nm; blue fluorescent protein (BFP) by excitation at 405 nm and monitoring at 450/50 nm; m-Cherry fluorescent protein by excitation at 561 and monitoring at 610/20; *CHOP::GFP* by excitation at 488 nm and monitoring at 530/30 nm; *XBP1s::Turquoise* by excitation at 405 nm and monitoring at 450/50 nm. Data were processed using FlowJo and statistical analysis using Prism8 (GraphPad).

The sensitivity to UPR induction in CHO-K1 S21 cells bearing *CHOP::GFP* and *XBP1s::Turquoise* reporters was analyzed after transient transfection with 1 μg sgRNA-mCherry-Cas9 encoding plasmids, targeting *SURF4, LMAN1* and *HSPA5* (BiP protein). Each gene was targeted with two different sgRNA and four days after transfection cells were analyzed by flow cytometry.

Whole genome CRISPR screen

High-throughput screen was carried out as previously described (Shalem et al., 2014) using a Chinese hamster knockout CRISPR-Cas9 library containing 125,030 sgRNAs targeting 20,680 genes (most with 6 guides per gene) as well as 1,239 non-targeting sgRNAs as a negative control cloned into the lentiviral sgRNA expression vector pKLV-U6gRNA(BbsI)-PGKpuro2ABFP as described (Harding et al., manuscript in preparation). Approximately 2.1×10^8 CHO-K1 Tet-on_α1AT^{H334D}_Cas9 cells were infected at a multiplicity of infection (MOI) of 0.3, to favor infection with a single viral particle/cell. Two days post infection, cells were puromycin-selected (8 μg/ml) for 7 days to obtain BFP-positive (sgRNA) cells and were maintained at > 450x coverage at all times. Expression of α1AT was induced with 10 ng/ml doxycycline for 24 hr. Afterward, the cells were fixed and permeabilized for intracellular staining of α1AT polymers. Approximately 6.6×10^7 Mab2C1-stained fixed cells were subjected to FACS and collected in 3 bins according to their fluorescence intensity at 670 nm (Mab2C1): ‘brightest’ (~2% of total sorted), ‘medium-bright’ (~4.5% of total), and ‘dull’ (~10% of total)

as shown in Figure 1B. The first round of enrichment was carried on by extracting the genomic DNA of the 'brightest'-binned fixed cells (~1.3x10^6 cells) and recovering by PCR a 220bp fragment containing the sgRNA-bearing region (oligonucleotides 2182 and 1758). The amplicon was ligated into the parental lentiviral backbone (UK1789) to generate derivative enriched library 1 (Lib$_1$). The same infection-FACS procedure described above was performed to infect 2 × 10^7 parental CHO-K1 Tet-on_α1AT^{H334D}_Cas9 cells with the new derivative Lib$_1$. After FACS sorting, approximately 6.8 × 10^6 'brightest'-binned fixed cells (~2% of total sorted) were recovered and genomic DNA was extracted to generate a second derivative enriched library 2 (Lib$_2$) that was used for a second round of enrichment to infect 2 × 10^7 parental CHO-K1 Tet-on_α1AT^{H334D}_Cas9 cells. In each round an equal number of infected, untreated cells (no doxycycline) or uninfected, doxycycline-treated cells were passed without sorting as a control group.

Genomic DNA from fixed, enriched, and sorted populations as well as fixed, unsorted libraries was extracted from ~1-3 × 10^6 and ~3.6 × 10^7 cells respectively, by incubation in proteinase K solution [100 mM Tris-HCl pH 8.5, 5 mM EDTA, 200 mM NaCl, 0.25% SDS, 0.2 mg/ml Proteinase K] overnight at 50°C. To reverse formaldehyde crosslinks, samples were supplemented with 500 mM NaCl and incubated at 65°C for 16 hr. Integrated sgRNA sequences were amplified by nested PCR and the adaptors for Illumina sequencing (HiSeq4000) were introduced at the final amplification round using oligonucleotides 1759-1769 (Table S3). Quality and purity of the PCR product were assessed by bioanalyzer (Agilent). Downstream analysis to obtain sgRNA read counts, gene rankings, and statistics were obtained using the MAGeCK computational software (Li et al., 2014). Gene ontology analyses were performed using Metascape software with default parameters (Zhou et al., 2019).

Validation of candidate genes

Two individual sgRNAs designed in the library targeting exon regions of *Cricetulus griseus LMAN1*, *SURF4* and *SEC23B* were cloned into the pSpCas9(BB)-2A-mCherry plasmid (UK1610) as previously reported (Ran et al., 2013). Cells were transfected with 1 μg of sgRNA/Cas9 plasmids UK2501-UK2506) using Lipofectamine LTX (Thermofisher). Forty-eight hours after transfection, mCherry-positive cells were individually sorted into 96-well plates using a MoFlo Cell Sorter (Beckman Coulter). The presence of frame-shift-causing insertion/deletions in both alleles of the obtained clones was achieved by capillary electrophoresis on a 3730xl DNA analyzer (Applied Biosystems) and amplifying the targeted region by PCR using a gene-specific 5′ 6-carboxyfluorescein (FAM)-labeled oligonucleotides (Hjelm et al., 2010). The knockouts were confirmed by Sanger sequencing and immunoblotting. Genomic information of the clones used in this study is provided in Table S2.

Mammalian cell lysates, sandwich ELISA, and immunoblotting

Cells were lysed in Nonidet lysis buffer [150 mM NaCl, 50 mM Tris-HCl pH 7.5, 1% Nonidet P-40] supplemented with protease inhibitor mixture (Roche) for 20 min on ice. To quantify polymer and total levels of intracellular α1AT, cell lysates were analyzed by sandwich ELISA using the polymer-specific Mab2C1 and a monoclonal antibody that recognizes all α1AT conformers (Mab3C11) (Tan et al., 2015) respectively. Briefly, high binding surface COSTAR 96-well plates (Corning) were coated overnight with purified rabbit polyclonal antibody against total α1AT at 2 μg/ml in PBS. After washing with PBS containing 0.9% NaCl and 0.05% Tween-20, the plates were blocked for 1 hr in blocking buffer (PBS containing 0.25% BSA and 0.05% Tween-20). Samples and standard curves were diluted in blocking buffer and incubated for 2 hr with the primary antibodies, Mab2C1 or Mab3C11. Anti-mouse IgG horseradish peroxidase-labeled antibody was used as a secondary antibody and incubated for 1 hr. The reaction was developed with TMB liquid substrate for 10 min in the dark, and the reaction was stopped with 1 M H_2SO_4. Absorbance was read at 450 nm on a microplate reader. For immunoblots, SDS sample buffer was added to the lysates and proteins were denatured by heating at 70°C for 10 min and separated on 10%–12% SDS-PAGE gels and transferred onto PVDF membranes prior to immunodetection. Cyclophilin B and actin were detected as loading controls. To detect the multi-pass transmembrane protein SURF4, samples were incubated at 37°C for 15 min. Native-PAGE (4.5% stacking gel and a 7.5% separation gel) was performed to separate and identify α1AT monomers and polymers. Membranes were scanned using an Odyssey near infrared imager (LI-COR) and signals were quantified with Fiji (ImageJ).

[^{35}S] metabolic labeling and immunoprecipitation

Cells were starved in Methionine/Cysteine-free DMEM (21013024, GIBCO) for 1 hr, pulsed with 100 μCi/well [^{35}S]methionine/cysteine (Expre^{35}S Protein Labeling Mix) and harvested or chased in DMEM containing 200 mM methionine and cysteine and 10% dialysed FBS. After the chase, culture media were collected and cells harvested on ice in Nonidet lysis buffer supplemented with protease inhibitor mixture (Roche). Culture media and cell lysates were precleared and α1AT was immunoprecipitated with a α1AT polyclonal antibody (total) or the Mab2C1 (polymer-specific) by splitting each sample in two equal parts. Radiolabelled proteins were recovered in 2 × SDS-PAGE loading buffer, separated on 10% SDS-PAGE gels, detected by autoradiography with a Typhoon biomolecular imager (GE Healthcare) and quantified using Fiji (ImageJ).

Cross-linking and co-immunoprecipitation

CHO-K1 Tet-on cells expressing α1AT^{WT} or α1AT^{H334D} were grown in 10-cm dishes and transfected with either a 7 × His- or FLAG-tagged SURF4 (UK2622 and UK2549) for 6 hr. Afterward, medium was exchanged against medium supplemented with 500 ng/ml doxycycline and cells were further incubated for 20 hr. Cross-linking was performed following a previously-published protocol (Zlatic et al., 2010) with modifications. Cells were washed twice with PBS/Ca/Mg solution (PBS containing 0.1 mM $CaCl_2$ and 1 mM $MgCl_2$)

and incubated for 2 hr on ice with 1 mM dithiobis(succinimidyl propionate) (DSP, reversible crosslinker) diluted in pre-warmed (37°C) PBS/Ca/Mg solution. The DSP-containing solution was removed and the residual DSP was quenched for 15 min with PBS/Ca/Mg solution supplemented with 20 mM Tris-HCl pH 7.4. Cells were washed with PBS/Ca/Mg and lysed in Nonidet lysis buffer. A post-nuclear supernatant was prepared by centrifugation at 20,000 × *g* at 4°C for 15 min, and then cleared again at 20,000 × *g* for 5 min. For immunoprecipitation of FLAG-SURF4, cell lysates (750 μg total protein) were precleared with empty agarose beads and then incubated with anti-FLAG-M2 agarose affinity beads (Sigma) with rotation overnight at 4°C. Beads were washed four times with RIPA buffer [50 mM Tris-HCl pH 8, 150 mM NaCl, 1% Triton X-100, 0.5% sodium deoxycholate, 0.1% SDS]. Bound proteins were eluted by addition of 2 × SDS sample buffer (without DTT) and shacking at 37°C for 15 min to avoid aggregation of SURF4. Eluted proteins were recovered at 2,800 × *g* for 5 min, 50 mM DTT was added and samples were further incubated at 37°C for 10 min. For pulldowns of 7xHis-SURF4, cell lysates were incubated in denaturing binding buffer (8 M Urea, 10 mM imidazole) containing protease inhibitors. Cell lysates were loaded onto Ni-NTA agarose beads (QIAGEN) and incubated with orbital rotation overnight at RT. The beads were washed in denaturing washing buffer containing 150 mM NaCl, 50 mM Tris, 8 M Urea. Over the three independent experiments different concentrations of imidaole were used (10, 20 and 30 mM, respectively) to successively increase stringency of the wash step. Beads were then suspended in elution buffer [8 M Urea, 2% SDS, 50 mM DTT, 4 mM EDTA]. Equal volumes of the samples were loaded on 12% SDS-PAGE gels. Samples of the normalized cell lysates (15 μg) were loaded as 'input' controls and bands were quantitated using Fiji (ImageJ).

Confocal microscopy

Cells were seeded on coverslips pretreated with 0.1 mg/ml poly-L-lysine (Sigma) in 12-well plates and then fixed with 4% paraformaldehyde for 30 min, followed by permeabilization with 0.1% Triton X-100 for 15 min. After 30 min blocking with PBS containing 10% BSA and 0.1% Triton X-100 the cells were co-stained with primary antibodies (Mab2C1 and anti-BiP) and the corresponding fluorescent secondary antibodies. Coverslips were mounted in FluorSave reagent (Calbiochem) containing 2% 1,4-diazabicyclo-[2.2.2]octane (Sigma). Imaging was performed on a Zeiss 710 confocal microscope using a 63x/1.4 oil immersion objective and diode, argon and HeNe lasers. The quantification of co-localization between both fluorescence channels (Pearson correlation coefficient) was quantified using Volocity software, version 6.3 (PerkinElmer).

QUANTIFICATION AND STATISTICAL ANALYSIS

All experiments were repeated at least three times unless otherwise indicated in each figure and legend. For all the statistical and quantitative analysis we used the predetermined functions in Graphpad Prism V8 software. Differences between groups were considered statistically significant if $p < 0.05$ (*, $p < 0.05$; **, $p < 0.01$; and ***, $p < 0.001$). All error bars represent mean ± SEM. All the details on statistical tests with 'n' values are indicated in the relevant figure legends and method sections.

Cell Reports, Volume *35*

Supplemental information

Cargo receptor-assisted endoplasmic reticulum export of pathogenic α1-antitrypsin polymers

Adriana Ordóñez, Heather P. Harding, Stefan J. Marciniak, and David Ron

Supplementary Figure 1 (Fig. S1)

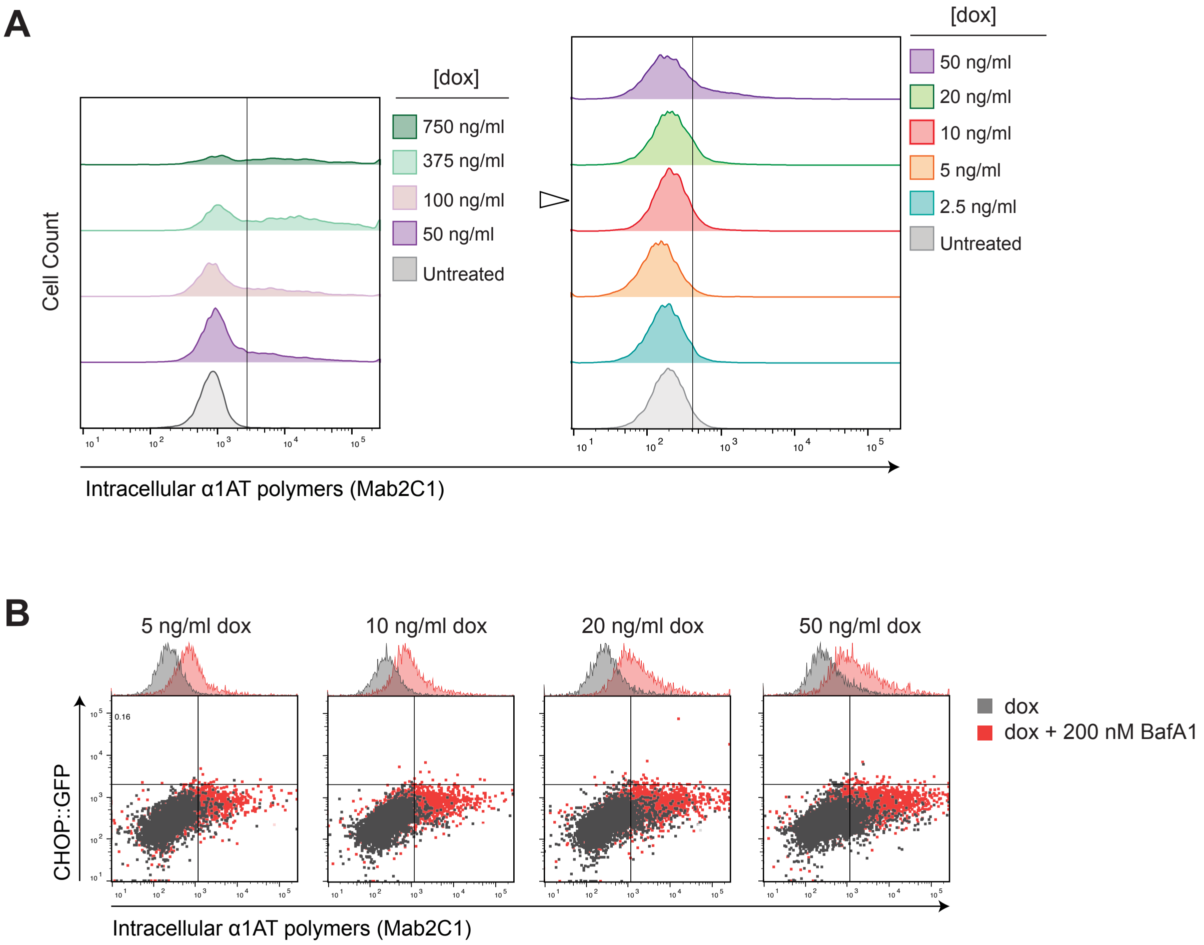

Fig. S1. Concentration-dependence of the response of CHO-K1 Tet-on cells to doxycycline and bafilomycinA1. Related to Figure 1.
(A) Flow cytometry analysis of the fluorescence intensity as a measure of intracellular α 1AT polymer levels (stained with Mab2C1) in CHO-K1 Tet-on_α1AT^{H334D}_Cas9 cells treated for 24 hrs with the indicated concentrations of doxycycline (dox). The left and right panels represent two independent experiments. The white arrowhead indicates the dox concentration used in the screen.
(B) Dual-channel flow cytometry of the UPR marker, *CHOP::GFP*, and intracellular levels of α1AT polymers in CHO-K1 Tet-on_α1AT^{H334D}_Cas9 cells treated for 24 hrs with the indicated concentration of dox in presence or absence of bafilomycinA1 (BafA1; 200 nM, added during the last 16 hrs). 5,000 cells were analyzed.

Supplementary Figure 2 (Fig. S2)

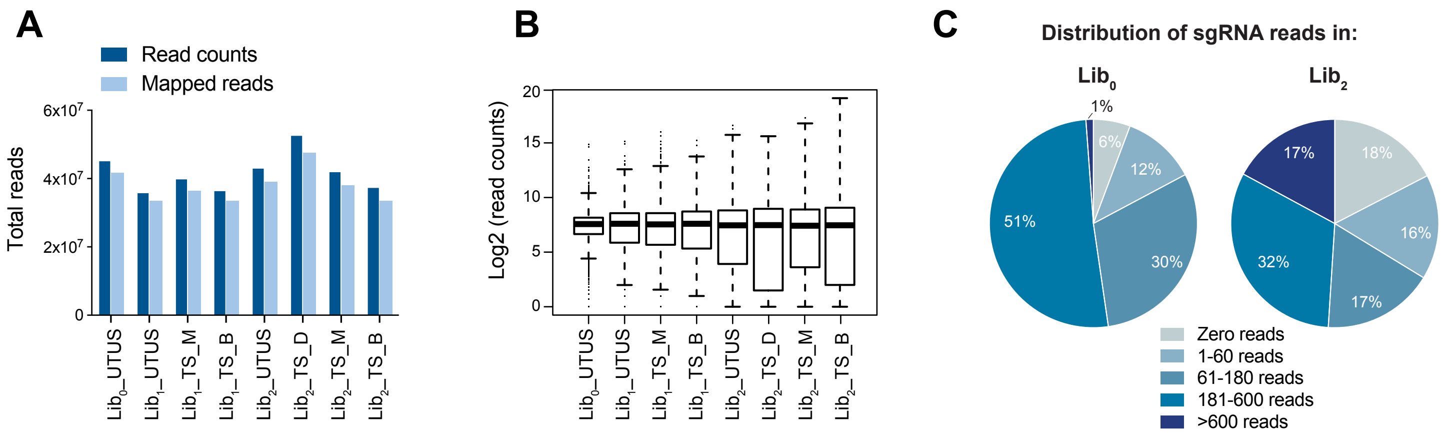

Fig. S2. Quality control data analysis of the CRISPR/Cas9 screen performed by MAGeCK. Related to Figure 2.

(A) Total read counts and reads mapped to the CHO library analysed by MAGeCK [UTUS: untreated (no doxycycline) and unsorted; TS: treated (plus doxycycline) and sorted; Lib_0: unenriched library) Lib_1: derivative enriched library 1; Lib_2: derivative enriched library 2; B: brightest; M: medium-bright; D: dull].

(B) Frequency distribution of sgRNA in each sample, showing the median-normalized read counts.

(C) Representation of sgRNAs in unsorted cells after infection with the unenriched genome-wide library (Lib_0) and enriched library (Lib_2) according to their read counts.

Supplementary Figure 3 (Fig. S3)

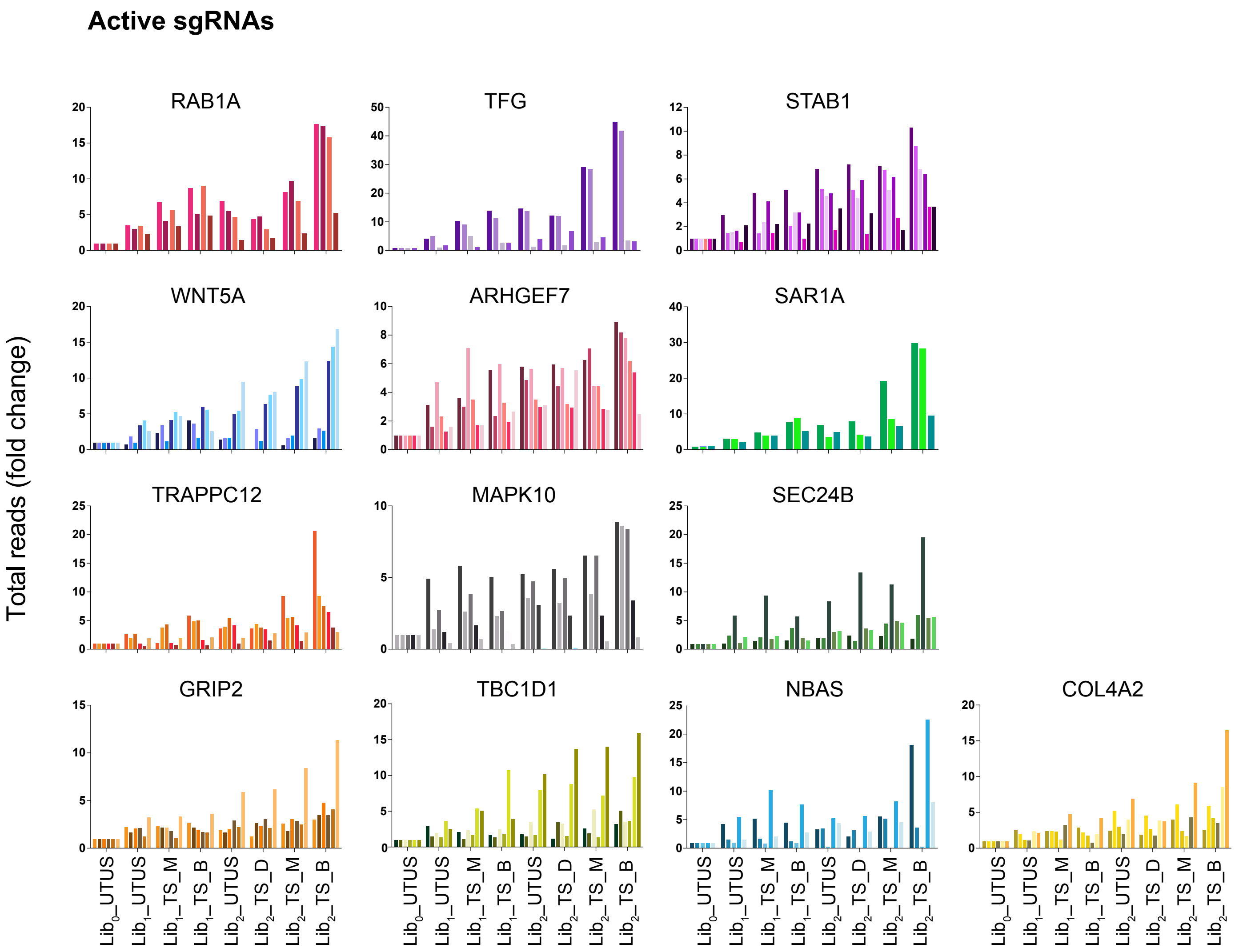

Fig. S3. The total reads counts for each active sgRNA targeting the remaining top 13 enriched genes included in the 'cargo loading into COPII-coated vesicle' cluster show an enrichment through the selection process. Related to Figure 2E.

Supplementary Figure 4 (Fig. S4)

A

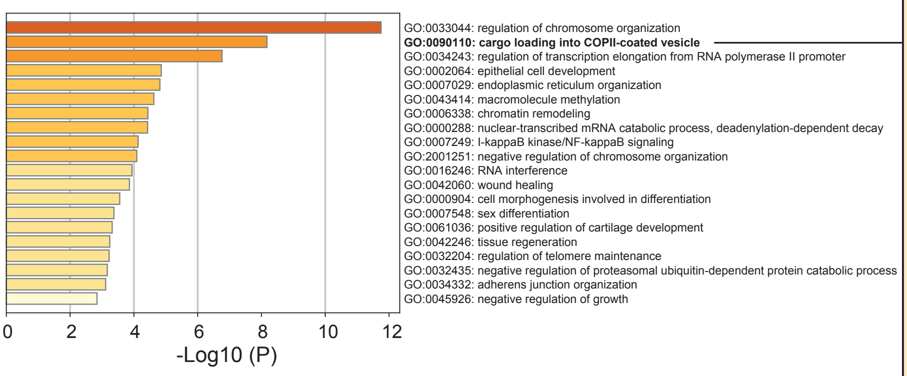

Gene	Description	Log2 (FC)
LMAN1	Lectin, mannose binding 1	*2.9204*
SEC23B	SEC23 homolog B, coat complex II component	*2.7843*
RAB1A	RAB1A, member RAS oncogene family	*2.3817*
TFG	Ttrafficking from ER to golgi regulator	*2.1409*
SAR1A	Secretion associated Ras related GTPase 1A	*2.032*
SURF4	Surfeit locus protein 4	*1.9554*
SEC24A	SEC24 homolog A, coat complex II component	*1.63*
WNT5A	Wnt family member 5A	*1.5715*
USE1	Unconventional SNARE in the ER 1	*1.5052*
BNIP1	BCL2 interacting protein 1	*1.429*
ALG12	ALG12 alpha-1,6-mannosyltransferase	*1.3852*
TRAPPC12	Trafficking protein particle complex 12	*1.3194*
PLEK	Pleckstrin	*1.1244*
IER3IP1	Immediate early response 3 interacting protein	*1.1125*
CASP3	Caspase 3	*1.0971*
STAB1	Stabilin 1	*1.0645*
SCFD2	Sec1 family domain containing 2	*0.97922*
COL4A2	Collagen type IV alpha 2 chain	*0.91986*
SEC24B	SEC24 homolog B, coat complex II component	*0.59458*
MON2	MON2 homolog, regulator of endosome-to-Golgi trafficking	*0.28108*

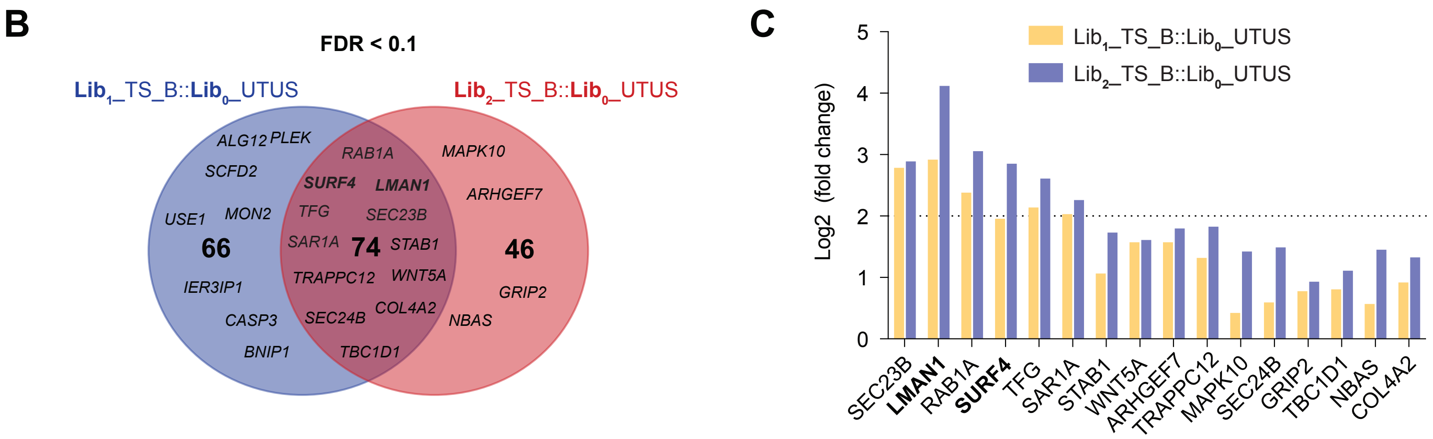

Fig. S4. Overlapping enrichment of specific sgRNAs targeting genes encoding components of the early secretory pathway in the first or second round of the CRISPR screen. Related to Figure 2.

(A) Gene ontology (GO) enrichment analysis of the top 140 hits identified in the CRISPR/Cas9 screen after the first round of enrichment (infection with Lib_1) and annotation of the 20 genes included in the GO term 'cargo loading into COPII-coated vesicle' indicating the corresponding Log2 (fold change) value for each gene.

(B) Venn diagram depicting unique and common upregulated top genes included in the 'cargo loading into COPII-coated vesicle' GO term, between the first (Lib_1) and second (Lib_2) round of enrichment after sorting.

(C) Histogram graph comparing the Log2 (fold change) values of the 16 genes included in the 'cargo loading into COPII-coated vesicle' cluster that were significantly enriched during the selection process after the first (Lib_1_TS_B::Lib_0_UTUS) and second round (Lib_2_TS_B::Lib_0_UTUS) of enrichment. Genes above the horizontal dashed line were enriched by a folded change of 4. LMAN1 and SURF4, the two cargo receptors selected for further investigation in our study, are in bold.

Supplementary Figure 5 (Fig. S5)

A

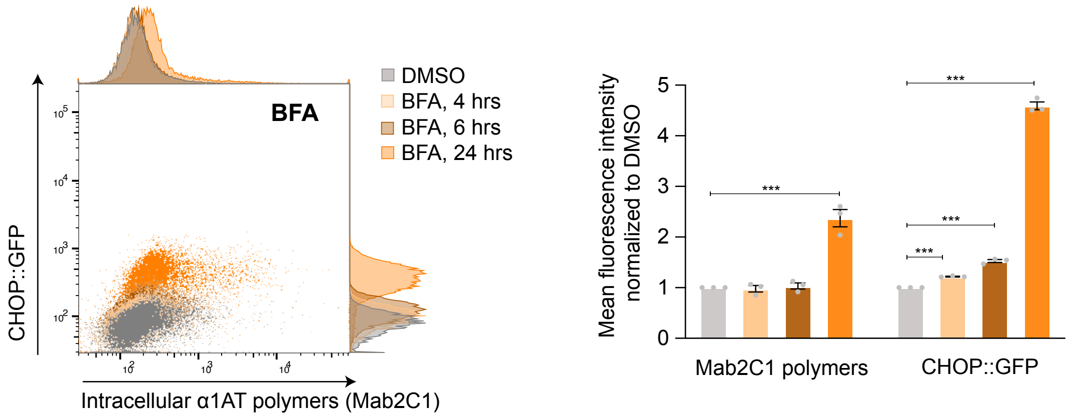

B

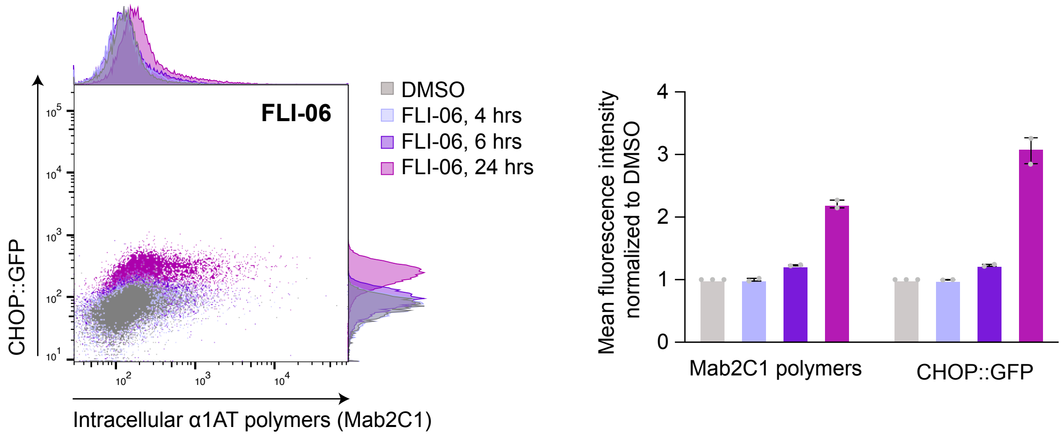

Fig. S5. Disruption of global endoplasmic reticulum-Golgi protein transport by brefeldin A (BFA) and FLI-06 increases the intracellular levels of α1-antitrypsin polymers and induces ER stress. Related to Figure 2.

(A) Dot plots of a representative dual-channel flow cytometry analysis of intracellular levels of α1AT polymers (Mab2C1) and *CHOP::GFP* reporter signal in CHO-K1 Tet-on_α1AT^{H334D} cells after treatment with brefeldin A (BFA). Cells were simultaneously induced with doxycycline (10ng/ml) and BFA (10 ug/ml) for 24 hrs, or induced with doxycycline for 24 hrs and BFA-treated for 4 and 6 hrs previous harvesting the cells. The bar graph shows the mean ± SEM of the Mab2C1-polymer and *CHOP::GFP* signal normalized to vehicle treated control cells (DMSO) from two independent experiments, one of them performed in duplicate (Unpaired t-test).

(B) As in "A" but plotting the intracellular levels of α1AT polymers and *CHOP::GFP* reporter signal after FLI-06 treatment (10 uM). The bar graph shows the mean ± SEM of one single experiment performed in duplicate.

Supplementary Figure 6 (Fig. S6)

A **Related to Fig. 5C**

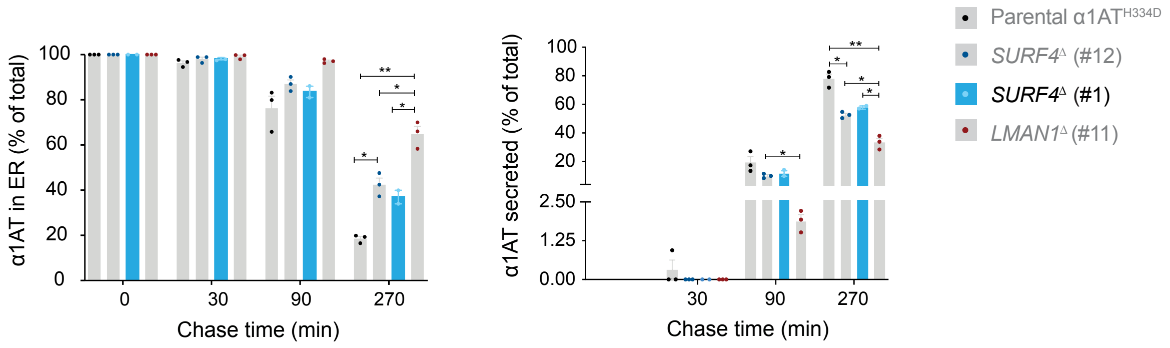

B **Related to Fig. 5D**

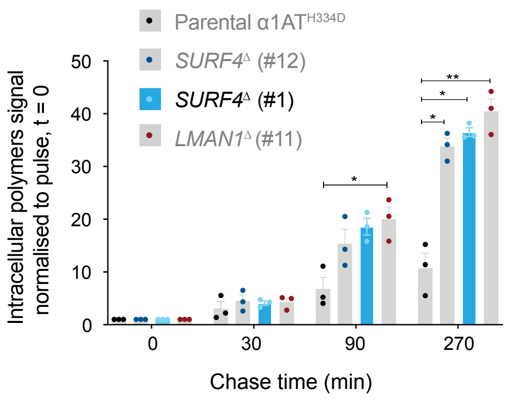

C **Related to Fig. 5E**

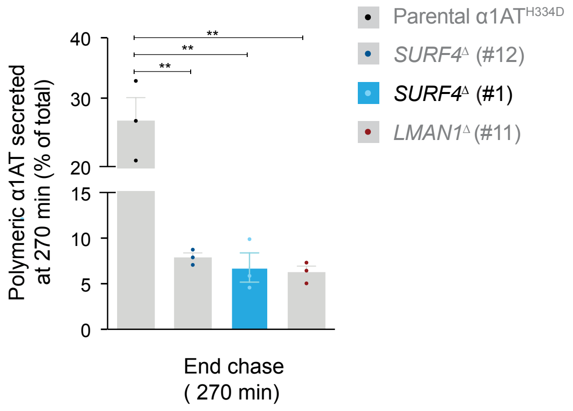

Fig. S6. Altered intracellular trafficking of α1-antitrypsin in an additional *SURF4*^{Δ} clone. Related to Figure 5.

Labelled α1AT was immunoprecipitated with a polyclonal antibody reactive with all α1AT forms or a monoclonal antibody selective for α1AT polymers from lysates of parental CHO-K1 Tet-on_α 1AT^{H334D} cells and their *SURF4*^{Δ} and *LMAN1*^{Δ} derivatives or from the culture media supernatant.

(A) Related to Fig. 5C. Plots of the percentage of α1AT retained in the ER (left panel) or secreted into the media (right panel) at the indicated times. The additional *SURF4* disrupted clone [*SURF4*^{Δ} (#1)] is highlighted in blue and the other three genotypes (previously shown in Fig. 5C) are coloured in grey.

(B) Related to Fig. 5D. Plot of the intracellular polymer signal normalized to polymer α1AT signal at pulse end (t = 0) at the indicated times. The additional *SURF4*^{Δ} (#1) clone is highlighted in blue.

(C) Related to Fig. 5E. Plot of the percentage of α1AT polymers present in the media at 270 min. The additional *SURF4*^{Δ} (#1) clone is highlighted in blue.

All quantitative plots show the mean ± SEM of two or three independent experiments; *p<0.05, **p<0.01. Two-way (in 'A' and 'B') or one-way ANOVA (in 'C') followed by Tukey's post-hoc multiple comparison test.

Supplementary Figure 7 (Fig. S7)

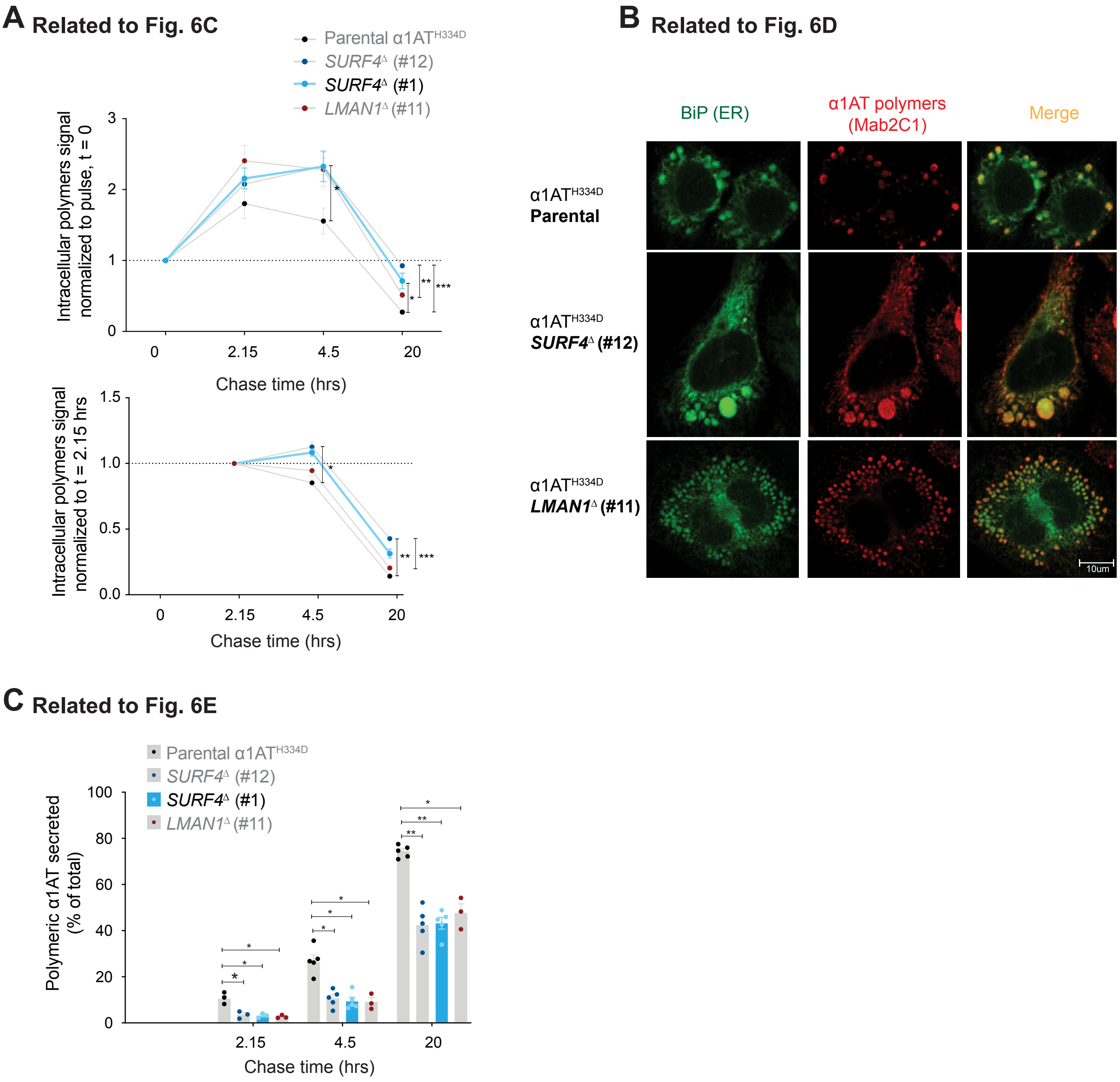

Fig. S7. SURF4 favours ER exit of α1-antitrypsin polymers in an additional *SURF4*$^{\Delta}$ clone. Related to Figure 6.

Labelled α1AT was immunoprecipitated with a monoclonal antibody selective for α1AT polymers from lysates of parental CHO-K1 Tet-on_α1AT^{H334D} cells and their *SURF4*$^{\Delta}$ and *LMAN1*$^{\Delta}$ derivatives or from the culture media supernatant.

(A) Related to Fig. 6C. Plots of the cell-associated α1AT polymer signal at the indicated times, normalized to the signal at pulse end [t = 0, (upper panel)] or at 2.15 hrs (bottom panel). The additional *SURF4* disrupted clone [*SURF4*$^{\Delta}$ (#1)] is highlighted in blue and the other three genotypes (previously showed in Fig. 6C) are coloured in grey.

(B) Related to Fig. 6D. Representative confocal immunofluorescence microscopy images of α1AT polymers (Mab2C1, red) together with an ER marker (BiP, green) in fixed parental CHO-K1 Tet-on_α1AT^{H334D} cells and their *SURF4*$^{\Delta}$ (#12) and *LMAN1*$^{\Delta}$ (#11) derivatives clones. α1AT expression was induced with 500 ng/ml doxycycline for 24 hrs.

(C) Related to Fig. 6E. Percentage of α1AT polymers present in the media at the indicated times. The additional *SURF4*$^{\Delta}$ (#1) clone is highlighted in blue.

All quantitative plots show the mean ± SEM of three to five independent experiments; *p<0.05, **p<0.01, ***p<0.001, ****p<0.0001. Two-way ANOVA test followed by Tukey's post-hoc multiple comparison test.

Supplemental Tables

Table S2: List of clones generated in this study. Related to STAR Methods.

Gene targeting	Cell line	Clone	Exon	Allele	Amino acid sequence (number shows amino acid position at which insert/deletion occurred)
SURF4	CHO-K1 Tet-on α1AT^{H334D}_CHOP::GFP	#12	5	1	….VTMR149in*
				2	….VTMR149in*
		#1	2	1	….DGIR44delSGVSNVTILTLPGAVATCWPHPLCSSTSWDS*
				2	∆W45-Q47
	CHO-K1 Tet-on α1AT^{WT}_CHOP::GFP	#21	5	1	….VTMR149in*
				2	….VTMR149inLRGHPDRQAEGDQGWPPALRLVRAPLSSTCNLEEGSCWS*
LMAN1	CHO-K1 Tet-on α1AT^{H334D}_CHOP::GFP	#11	9	1	….VSSL375delRRDLQERSGDPRAAWAGLSTGTRYSCENPA*
				2	….VSSL375delKKRSPGEERGPQGSLGRSLNRN*
		#14	11	1	….QHPG433indelVYETTSALHGHQRAPACREERY*
				2	….QHPG433inWHVVALAPVLVASGQHLVVDGVVLQKALRHDMVHIVVGQPVDVQFLVHVHIPPILQVVQVQLLVLQLGVFHGVFFQDLAAQLFDAXXDPXXSLAAVLLSRRRL*
	CHO-K1 Tet-on α1AT^{WT}_CHOP::GFP	#8	9	1	….VSSL375delRSPGEERGPQGSLGRSLNRN*
				2	….VSSL375delKKRSPGEERGPQGSLGRSLNRN*
SEC23B	CHO-K1 Tet-on α1AT^{H334D}_CHOP::GFP	#1	7	1	….KTP313delIPGTILKKIMHGS*
				2	….KTP313delIPGTILKKIMHGS*

Table S3: List of sgRNAs and oligonucleotides used in this study. Related to STAR Methods.

Lab ID	Name	Sequence 5' – 3'	Comment	Reference
2486	cgLman1_g1_e11_1S	CACCGCTCATAGACGCCTG CAGAGC	CRISPR-Cas9 guide targeting chinese hamster Lman1 exon 11	This study
2487	cgLman1_g1_e11_2A S	AAACGCTCTGCAGGCGTCT ATGAGC	CRISPR-Cas9 guide targeting chinese hamster Lman1 exon 11	This study
2488	cgLman1_g2_e9_1S	CACCGCCTGGAGATCTCTT CTGTCA	CRISPR-Cas9 guide targeting chinese hamster Lman1 exon 9	This study
2489	cgLman1_g2_e9_2A S	AAACTGACAGAAGAGATCT CCAGGC	CRISPR-Cas9 guide targeting chinese hamster Lman1 exon 9	This study
2490	cgSurf4_g1_e5_1S	CACCGCTTAGGGGAGCTCT CACGCA	CRISPR-Cas9 guide targeting chinese hamster Surf4 exon 5	This study
2491	cgSurf4_g1_e5_2AS	AAACTGCGTGAGAGCTCCC CTAAGC	CRISPR-Cas9 guide targeting chinese hamster Surf4 exon 5	This study
2492	cgSurf4_g2_e2_1S	CACCGCATCCGCATGTGGT TTCAG	CRISPR-Cas9 guide targeting chinese hamster Surf4 exon 2	This study
2493	cgSurf4_g2_e2_2AS	AAACCTGAAACCACATGCG GATGC	CRISPR-Cas9 guide targeting chinese hamster Surf4 exon 2	This study
2494	cgSec23b_g1_e7_1S	CACCGATATCGTGCCAGGA ACGAAT	CRISPR-Cas9 guide targeting chinese hamster Sec23b exon 7	This study
2495	cgSec23b_g1_e7_2A S	AAACATTCGTTCCTGGCAC GATATC	CRISPR-Cas9 guide targeting chinese hamster Sec23b exon 7	This study
2496	cgSec23b_g2_e13_1 S	CACCGCAGTCTTGATGGCA CGGCT	CRISPR-Cas9 guide targeting chinese hamster Sec23b exon 13	This study
2497	cgSec23b_g2_e13_2 AS	AAACAGCCGTGCCATCAAG ACTGC	CRISPR-Cas9 guide targeting chinese hamster Sec23b exon 13	This study
2547	cgSurf4_e2_1S	ACCAAGCAGTACCTGCCTC A	for sequencing CRISPR mutants made in the cgSurf4 locus	This study
2548	cgSurf4_e2_2AS	ACACAAAGGATGAGGCCAA C	for sequencing CRISPR mutants made in the cgSurf4 locus	This study
2549	cgSurf4_e5_1S	GAGGTTTGCTGCTGCTCTT G	for sequencing CRISPR mutants made in the cgSurf4 locus	This study
2550	cgSurf4_e5_2AS	AGCTGGCATCAAAGTGAAG G	for sequencing CRISPR mutants made in the cgSurf4 locus	This study
2516	cgLman1_e11_1S	GAACTCCATGAGTGAAACA GTCC	for sequencing CRISPR mutants made in the cgLman1 locus	This study
2517	cgLman1_e11_2AS	ATGTTGCGCTGAGCAAGG	for sequencing CRISPR mutants made in the cgLman1 locus	This study
2518	cgLman1_e9_1S	CGATCGCGAGCTAAGACAA G	for sequencing CRISPR mutants made in the cgLman1 locus	This study
2519	cgLman1_e9_2AS	CTGGAGCATTTTGAGGGAA C	for sequencing CRISPR mutants made in the cgLman1 locus	This study
2528	cgSec23b_e7_1S	GGATCATGCTGTTCACTGG A	for sequencing CRISPR mutants made in the cgSec23b locus	This study
2529	cgSec23b_e7_2AS	AGTGACAGCTGGAATCCAC A	for sequencing CRISPR mutants made in the cgSec23b locus	This study
2182	sgRNA_outer_MluI_s hort_F	CAGCAGAGATCCAGTTTGG TTAGTACC	primer for PCR of pKLV CHO_CRISPR library for recloning in UK1789	This study
1432	P5-sgRNA_inner_F	AATGATACGGCGACCACCG AGATCTACACTCTCTTGTGG AAAGGACGAAACACCG	primer for barcoding and adapting lentiGuide PCR products from CRISPR library screening for NGS	Harding et al., 2019
1434	sgRNA_outer_short_ F	GCTTACCGTAACTTGAAAGT ATTTCG	primer for barcoding and adapting lentiGuide PCR products from CRISPR library screening for NGS	Harding et al., 2019
1435	Illumina-sgRNA_seq	ACACTCTCTTGTGGAAAGG ACGAAACACCG	PAGE purified primer for NGS of PCR products from CRISPR library screening	Harding et al., 2019
1758	sgRNA_outer_short_ R2	GAATGTGTGCGAGGCCAGA G	primer for 1st round PCR of pKLV CHO_CRISPR library for NGS sequencing	Harding et al., 2019

1759	pKLV_NEBNXT01	CAAGCAGAAGACGGCATACGAGATCGTGATGTGACTGGAGTTCAGACGTGTGCTCTTCCGATCTGAGGCCACTTGTGTAGCGCCAAG	primer for barcoding and adapting pKLV CHO_CRISPR PCR products for NGS	Harding et al., 2019
1760	pKLV_NEBNXT02	CAAGCAGAAGACGGCATACGAGATACATCGGTGACTGGAGTTCAGACGTGTGCTCTTCCGATCTGAGGCCACTTGTGTAGCGCCAAG	primer for barcoding and adapting pKLV CHO_CRISPR PCR products for NGS	Harding et al., 2019
1761	pKLV_NEBNXT03	CAAGCAGAAGACGGCATACGAGATTGCCTAAGTGACTGGAGTTCAGACGTGTGCTCTTCCGATCTGAGGCCACTTGTGTAGCGCCAAG	primer for barcoding and adapting pKLV CHO_CRISPR PCR products for NGS	Harding et al., 2019
1762	pKLV_NEBNXT04	CAAGCAGAAGACGGCATACGAGATTGGTCAGTGACTGGAGTTCAGACGTGTGCTCTTCCGATCTGAGGCCACTTGTGTAGCGCCAAG	primer for barcoding and adapting pKLV CHO_CRISPR PCR products for NGS	Harding et al., 2019
1763	pKLV_NEBNXT05	CAAGCAGAAGACGGCATACGAGATCACTGTGTGACTGGAGTTCAGACGTGTGCTCTTCCGATCTGAGGCCACTTGTGTAGCGCCAAG	primer for barcoding and adapting pKLV CHO_CRISPR PCR products for NGS	Harding et al., 2019
1764	pKLV_NEBNXT06	CAAGCAGAAGACGGCATACGAGATTATTGGCGTGACTGGAGTTCAGACGTGTGCTCTTCCGATCTGAGGCCACTTGTGTAGCGCCAAG	primer for barcoding and adapting pKLV CHO_CRISPR PCR products for NGS	Harding et al., 2019
1765	pKLV_NEBNXT07	CAAGCAGAAGACGGCATACGAGATTGATCTGGTGACTGGAGTTCAGACGTGTGCTCTTCCGATCTGAGGCCACTTGTGTAGCGCCAAG	primer for barcoding and adapting pKLV CHO_CRISPR PCR products for NGS	Harding et al., 2019
1766	pKLV_NEBNXT08	CAAGCAGAAGACGGCATACGAGATTTCAAGTGTGACTGGAGTTCAGACGTGTGCTCTTCCGATCTGAGGCCACTTGTGTAGCGCCAAG	primer for barcoding and adapting pKLV CHO_CRISPR PCR products for NGS	Harding et al., 2019
1767	pKLV_NEBNXT09	CAAGCAGAAGACGGCATACGAGATTCTGATCGTGACTGGAGTTCAGACGTGTGCTCTTCCGATCTGAGGCCACTTGTGTAGCGCCAAG	primer for barcoding and adapting pKLV CHO_CRISPR PCR products for NGS	Harding et al., 2019
1768	pKLV_NEBNXT10	CAAGCAGAAGACGGCATACGAGATAAGCTAGTGACTGGAGTTCAGACGTGTGCTCTTCCGATCTGAGGCCACTTGTGTAGCGCCAAG	primer for barcoding and adapting pKLV CHO_CRISPR PCR products for NGS	Harding et al., 2019
1769	pKLV_NEBNXT11	CAAGCAGAAGACGGCATACGAGATTGTAGCCGTGACTGGAGTTCAGACGTGTGCTCTTCCGATCTGAGGCCACTTGTGTAGCGCCAAG	primer for barcoding and adapting pKLV CHO_CRISPR PCR products for NGS	Harding et al., 2019
2606	cgSurf4_exon4_6FAM_2AS	[6FAM]AGCTGGCATCAAAGTGAAGG	oligo 2550 with 5'-[6FAM] for screening for efficient CRISPRs	This study
2607	cgSurf4_exon1_6FAM_2AS	[6FAM]ACACAAAGGATGAGGCCAAC	oligo 2548 with 5'-[6FAM] for screening for efficient CRISPRs	This study
2665	cgLman1_exon10_6FAM_2AS	[6FAM]ATGTTGCGCTGAGCAAGG	oligo 2517 with 5'-[6FAM] for screening for efficient CRISPRs	This study
2666	cgLman1_exon8_6FAM_2AS	[6FAM]CTGGAGCATTTTGAGGGAAC	oligo 2519 with 5'-[6FAM] for screening for efficient CRISPRs	This study
1402	EGFP_guide1_1S	CACCGGGCGAGGAGCTGTTCACCG	CRISPR-Cas9 guide targeting EGFP	This study
1403	EGFP_guide1_2AS	AAACCGGTGAACAGCTCCTCGCCC	CRISPR-Cas9 guide targeting EGFP	This study

Table S4: List of Recombinant DNA used in this study. Related to STAR Methods.

Lab ID	Plasmid name	Description	Reference
UK1610	pSpCas9(BB)-2A-mCherry	Modified pSpCas9(BB)-2A vector to express mCherry together with guide RNA & Cas9	Amin-Wetzel N et al., 2017
UK1700	pMD2.G	Addgene plasmid 12259, lentiviral packaging helper, (VSVG)	Unpublished, gift from Didier Trono
UK1701	psPAX2	Addgene plasmid 12260, next gen lentiviral packaging helper	Unpublished, gift from Didier Trono
UK1702	LentiGuide-puro	Addgene plasmid 52963	Sanjana et al., 2014, gift from Feng Zhang
UK1714	Lenti-Cas9	Lenti-Cas9 in which 2TA-blast sequence is removed to make a lenti-Cas9 without resistance selection marker	This study
UK1717	EGFPsgRNA_lentiGuide-Puro	Lentiviral vector expressing EGFP CRISPR guides without expression of Cas9	This study
UK1789	pKLV-U6gRNA(BbsI)-PGKpuro2ABFP	Addgene 50946, BFP-2A-Puro tagged gRNAvector	Koike-Yusa et al., 2014, gift from Kosuke Yusa
UK1857	cgHSPA5_g1_pSpCas(BB)-2A-mCherry	mCherry-tagged CRISPR plasmid (UK1610) for targeting hamster HSPA5 (BiP)	Preissler et al., 2017
UK1858	cgHSPA5_g2_pSpCas(BB)-2A-mCherry	mCherry-tagged CRISPR plasmid (UK1610) for targeting hamster HSPA5 (BiP)	Preissler et al., 2017
UK2561	pKLV-CHO_libA-PGKpuro2ABFP (Library0)	CHO CRISPR KO library of 125030 selected guides for whole genome CRISPR screening	Unpublished
UK2321	pKLV-α1AT derivative enriched CHO library1 (MluI_BamHI)-PGKpuro2ABFP	CHO CRISPR KO derivative library 1 (Lib1) for α1AT polymer enrichment_Brightest population-After first sorting	This study
UK2378	pKLV-α1AT derivative enriched CHO library2 (MluI_BamHI)-PGKpuro2ABFP	CHO CRISPR KO derivative library 2 (Lib2) for α1AT polymer enrichment_Brightest population-After second sorting	This study
UK2501	cgLman1_g1_exon 11_pSpCas9(BB)-2A-mCherry	mCherry-tagged CRISPR plasmid (UK1610) targeting cgLMAN1_guide 1	This study
UK2502	cgLman1_g2_exon 9_pSpCas9(BB)-2A-mCherry	mCherry-tagged CRISPR plasmid (UK1610) targeting cgLMAN1_guide 2	This study
UK2503	cgSurf4_g1_exon 5_pSpCas9(BB)-2A-mCherry	mCherry-tagged CRISPR plasmid (UK1610) targeting cgSURF4_guide 1	This study
UK2504	cgSurf4_g2_exon 2_pSpCas9(BB)-2A-mCherry	mCherry-tagged CRISPR plasmid (UK1610) targeting cgSURF4_guide 2	This study
UK2505	cgSec23b_g1_exon 7_pSpCas9(BB)-2A-mCherry	mCherry-tagged CRISPR plasmid (UK1610) targeting cgSEC23b_guide 1	This study
UK2506	cgSec23b_g2_exon 13_pSpCas9(BB)-2A-mCherry	mCherry-tagged CRISPR plasmid (UK1610) targeting cgSEC23b_guide 2	This study
UK2549	FLAG-tagged SURF4 [pNLF-FLAG-SURF4-puro)	FLAG-tagged SURF4 [pNLF-FLAG-SURF4-puro)	Emmer et al., 2018, gift from David Ginsburg
UK2622	pNLF-H7-SURF4-puro	Mammalian expression plasmid 7xHis N-term tagged SURF4	This study
